# Copy Number Analysis Identifies Novel Interactions Between Genomic Loci in Ovarian Cancer

**DOI:** 10.1371/journal.pone.0011408

**Published:** 2010-09-10

**Authors:** Kylie L. Gorringe, Joshy George, Michael S. Anglesio, Manasa Ramakrishna, Dariush Etemadmoghadam, Prue Cowin, Anita Sridhar, Louise H. Williams, Samantha E. Boyle, Nozomu Yanaihara, Aikou Okamoto, Mitsuyoshi Urashima, Australian Ovarian Cancer Study, Gordon K. Smyth, Ian G. Campbell, David D. L. Bowtell

**Affiliations:** 1 Victorian Breast Cancer Research Consortium (VBCRC) Cancer Genetics Laboratory, Peter MacCallum Cancer Centre, East Melbourne, Australia; 2 Department of Pathology, University of Melbourne, Parkville, Australia; 3 Cancer Genetics and Genomics Laboratory, Peter MacCallum Cancer Centre, East Melbourne, Australia; 4 Department of Biochemistry, University of Melbourne, Parkville, Australia; 5 Murdoch Children's Research Institute, Royal Children's Hospital, Parkville, Australia; 6 Jikei University School of Medicine, Tokyo, Japan; 7 Bioinformatics Division, Walter and Eliza Hall Institute of Medical Research, Parkville, Australia; Georgia Institute of Technology, United States of America

## Abstract

Ovarian cancer is a heterogeneous disease displaying complex genomic alterations, and consequently, it has been difficult to determine the most relevant copy number alterations with the scale of studies to date. We obtained genome-wide copy number alteration (CNA) data from four different SNP array platforms, with a final data set of 398 ovarian tumours, mostly of the serous histological subtype. Frequent CNA aberrations targeted many thousands of genes. However, high-level amplicons and homozygous deletions enabled filtering of this list to the most relevant. The large data set enabled refinement of minimal regions and identification of rare amplicons such as at 1p34 and 20q11. We performed a novel co-occurrence analysis to assess cooperation and exclusivity of CNAs and analysed their relationship to patient outcome. Positive associations were identified between gains on 19 and 20q, gain of 20q and loss of X, and between several regions of loss, particularly 17q. We found weak correlations of CNA at genomic loci such as 19q12 with clinical outcome. We also assessed genomic instability measures and found a correlation of the number of higher amplitude gains with poorer overall survival. By assembling the largest collection of ovarian copy number data to date, we have been able to identify the most frequent aberrations and their interactions.

## Introduction

Epithelial ovarian cancer (EOC) is one of the deadliest malignancies, with high recurrence and poor survival rates [1]. The genetic aberrations observed in EOC are highly complex, comprising frequent aneuploidy and multiply rearranged chromosomes [2], [3]. The heterogeneity of copy number alterations (CNA) observed in EOC has made it difficult for small studies to be able to accurately identify the true frequency of the less common CNAs or to reproducibly identify CNAs that correlate with clinical parameters. A small sample size also makes it difficult to identify CNAs that co-exist or are mutually exclusive, which is a prerequisite to identify any common pathways that may be deregulated in EOC through alterations in gene copy number. The paradigm for mutually exclusive aberrations targeting the same pathway was set in colorectal tumours for *APC* and *CTNNB1* mutations [4] and extended in other examples such as exclusivity of *BRAF* and *KRAS* mutations [5]. Conversely, other genetic aberrations are more frequently observed in the same tumour than would have been expected by chance, suggesting a co-operative effect, for example the significant association of 11q13 and 8p12 amplicons in breast cancer [6]. In ovarian cancer, associations have been found between *CCNE1* and 12p amplification [7], and between *MYC* and 20q amplification [8] by fluorescence *in situ* hybridisation. Few studies have examined co-operativity or complementation of CNA on a genome-wide basis. Losses at 4q and 18q were found to be associated in one study [9] but this was not replicated in a recent analysis [10], which identified 7 CNA associations and 6 anti-correlations.

The presence of high level gene amplifications in ovarian cancer has been observed for some time, however most studies have been underpowered in sample size [10] or genomic resolution [11], [12] to accurately detect the frequency and target of these events. Similarly, few robust associations of CNA with clinical parameters such as survival have been identified [13], [14]. The detection of these CNA is relevant not only to the identification of tumour subgroups and the pathways affected in the tumours, but also to the targeting of molecular therapies in ovarian cancer. In this study we have brought together a large cohort of single nucleotide polymorphism (SNP) mapping array data to robustly annotate CNAs in serous and endometrioid ovarian cancers in order to identify the genes targeted by these genetic events and how these correlate with clinical parameters. In addition, we have assessed the interaction of CNA by evaluating their associations and anti-associations.

## Materials and Methods

### Peter MacCallum Cancer Centre (PMCC) data set: Tissue samples and DNA extraction

All samples were collected with the patient's informed consent and the study was approved by all the participating hospital Human Research Ethics Committees. Patients with ovarian cancer were identified through four primary sources between 1992 and 2006: a) 53 at hospitals in Southampton, UK, b) 141 through the Australian Ovarian Cancer Study, including 20 from the Westmead Gynaecological Oncology Tissue Bank, c) 15 through the PMCC Tissue Bank (Melbourne, Australia) and d) 41 from Jikei University (Tokyo, Japan). Pathology review was conducted from either formalin fixed, paraffin embedded tissue and/or fresh-frozen sections adjacent to the tissue from which DNA was extracted (n = 141) or through examination of the original diagnostic pathology reports (n = 109) (Table 1, Table S1).

**Table 1:** Summary of clinico-pathologic features of samples in the final data set.

				Grade^2^			Stage^2^			
Data set^1^	Age (median)	Subtype	Total	1	2	3	I	II	III	IV
50 K Australia	58	Serous	95	6	34	50	3		80	12
		Endometrioid	9	1	4	2	1	1	5	1
		Other	4			1			2	
250 K Japan	54	Serous	12	2			2	1	6	2
		Endometrioid	11		2		2		4	5
500 K Australia	64	Serous	17	1	5	11	1	6	9	1
		Endometrioid	10	1	4	5	3	1	3	2
SNP6 Australia	61	Serous	65	3	21	39	2	7	49	1
		Endometrioid	16		6	9	7	4	4	1
		Other	2			2		1	1	
SNP6 TCGA	60	Serous	157		7	145	2	4	118	30

1 Unique, successful samples only.

2 Only samples with known grade or stage shown.

All tissue samples were collected as fresh frozen material. A representative haematoxylin and eosin stained section was assessed and samples with >80% epithelial cells were used directly for DNA extraction from the whole tissue. For the remainder, needle or laser dissection was performed using 10 µm sections to obtain high percentage tumour epithelial cell component. DNA was extracted as previously described [14], [15]. Normal DNA extracted from blood lymphocytes was available for 106 patients.

### The Cancer Genome Atlas (TCGA) data set: Tissue samples and DNA extraction

Samples were collected as fresh frozen material from hospitals in the USA (n = 163). Tumour samples were assessed to be >80% of epithelial cells prior to DNA extraction from the whole tissue, as outlined [16]. Normal DNA extracted from blood lymphocytes was available for 161 patients. The results published here are in part based upon data generated by The Cancer Genome Atlas pilot project established by the NCI and NHGRI. Information about TCGA and the investigators and institutions who constitute the TCGA research network can be found at http://cancergenome.nih.gov.

### Copy number arrays

Samples were processed as previously described for Affymetrix Mapping arrays a) n = 108 50 K *Xba*I [14], GSE 13813 b) n = 27 250 K *Sty*I arrays c) n = 32 500 K arrays (250 K *Sty*I and 250 K *Nsp*I, [17]) d) n = 83 SNP6.0 (1.8 M probe sets [15], [18], GSE19539). When available, matching normal DNA was also analysed on the same array platform and in the same batch. TCGA SNP6.0 CEL files for 163 samples were downloaded from the Data Portal (http://tcga-data.nci.nih.gov/tcga/homepage.htm).

### Data pre-processing and analysis

All SNP Mapping arrays were first normalized using methods available in the R package “aroma.affymetrix” [19], including techniques to remove systematic biases introduced due to allelic cross talk, PCR fragment length bias and differences in GC content. DNA copy number was estimated probe set-wise by comparing the normalized signal from a tumour sample to data from normal lymphocyte DNA from the same patient, if available. On tumour samples for which matched normal tissue was not available, the average signal from all the normals generated in the same lab was used as reference. Quality control steps are described in Methods S1. Only the included samples are summarised in Table 1.

The circular binary segmentation method was used to segment the copy normalized data [20], [21]. Any probe sets within a CNA that was present in >5% of normal samples were excluded from the tumour analysis prior to segmentation to remove common copy number polymorphisms (CNP). Segments with fewer than 10 probe sets (SNP6) or 5 probe sets (500 K) were merged with the adjacent segment of closest copy number as previous QPCR analysis suggested that aberrations represented by few probes on these platforms may not be reliable [17]. In addition, we used Genomic Identification of Significant Targets in Cancer (GISTIC), which is a method that aggregates data over different tumours to try to differentiate between driver and passenger aberrations, combining prevalence and amplitude [22]. This technique was performed using a web-based interface (http://genepattern.broadinstitute.org) with CNA thresholds of ±0.3, a minimum of 10 markers and a q-value threshold of 0.25.

For hierarchical clustering, all tumours were assessed for the presence (“1”) or absence (“0”) of each GISTIC peak alteration (n = 89), where any overlap was considered as presence. Hierarchical clustering using average Euclidean clustering of the samples (n = 398) was performed using Partek Genomics Suite v.6.4 (Partek Inc., St. Louis, MO).

### Association between regions of aberrations

We undertook the analysis of association on the TCGA data set (for which we re-ran GISTIC) and then on the remaining samples. Two different methods were used to compute associations between regions of gain and loss. GISTIC results were summarized as a matrix X with tumours as rows and regions of aberrations as columns. For each tumour (t) and focal region of aberration (i), the measurement X[t,i] was 1 if the aberration was present for that tumour and 0 otherwise. A Poisson log-linear model was fit to the contingency table describing the aberration status. Statistical significance of the association was computed using a score test that yields a standard normal z-statistic [23]. This is equivalent to the square-root of the usual Pearson test statistic for independence, signed according to the direction of the association. The Benjamini and Hochberg method was used to correct for multiple testing [24].

Association between regions of aberrations was also tested using the Monte Carlo permutation test. Briefly, every column in matrix X was permuted independently (maintaining the number of entries in the columns to be the same). A score for association was computed using the permuted matrix as described for the parametric test above. The average rank obtained for every pair of regions from a large number of permutations was used to estimate the false discovery rate and the number of times a test statistic greater than or above the original test statistic was used to compute the p-value. Using a 5% false discovery rate the methods selected >98% of the same pairs of regions. We chose to use the first method described for region selection but both are reported.

### Analysis of expression correlations between associated copy number aberrations

We posited that the correlation between regions of aberrations should result in correlation of mRNA levels of the genes within the region. Affymetrix U133A array data was obtained for all samples from TCGA. For all associated regions above, four Pearson correlation tests were performed for the genes in the regions: a) correlation of copy number between Gene X in Region A and Gene Y in Region B, b) correlation between copy number and expression of Gene X in Region A, c) correlation between copy number and expression of Gene Y in Region B and d) correlation of expression between Gene X and Gene Y. All four tests had to be significant at p<0.05.

### Survival Associations

The Cox proportional hazard model was used to compute the association between regions of aberration detected by GISTIC and overall or progression free survival, correcting for multiple testing using the Benjamini-Hochberg method. To compute the survival association with pairs of regions, samples were classified into four groups based on the aberration status of the pairs of regions. Similarly, for the genomic measures, samples were binned into one of four groups based on data quartiles for each measure. Survival association with the groups thus identified was computed using the Cox proportional hazard model.

## Results

### Integration of copy number alterations from 398 ovarian carcinomas

We compiled high resolution copy number data from nearly 400 ovarian cancer samples representing two histological subtypes, serous and endometrioid (Table 1), 270 of which had matching normal lymphocyte DNA data. Data was compiled from multiple sources: high quality Affymetrix SNP6.0 Mapping Array “CEL” files were sourced through The Cancer Genome Atlas (TCGA, 157 cases) or were obtained at the Peter MacCallum Cancer Centre (83 cases [18]) SNP Mapping array data derived from lower resolution Affymetrix platforms including 108 cases assayed on 50 K *Xba*I arrays [14], 27 cases on 500 K arrays [15] and 23 cases on 250 K *Sty*I arrays obtained from Japan, were also included. Extensive quality control criteria were applied to all data sets (see Methods S1). Following normalisation of each data set, copy number alterations (CNA) were detected by circular binary segmentation [21]. We evaluated a number of possibilities for combining the datasets including cohort-specific thresholds (see Methods S1), however this made little difference to the final CNA pattern and a standard threshold of +/− 0.3 (log_2_) was applied universally as previously described by us [17] and others [10].

Comparison between the five sources of data showed a remarkable consistency of CNA across the genome, indicating a high degree of non-randomness to the CNA and equally importantly, an absence of significant array batch effects (Figure S1). The exception was the Japanese data set, which appeared to show a reduced number of alterations. However, a genome-wide test was conducted to identify regions aberrant at different frequencies between different platforms and could not identify any statistically significant regions after multiple testing correction.

We assessed the possibility of molecular subgroups within the combined cohort defined by copy number using hierarchical clustering (Figure S1). Only a single group of samples was distinguishable; these had few CNAs and tended to be low-grade samples or the Japanese samples, for which grade information was mostly not available. There were no other distinct clusters or major groupings attributable to histological subtype or grade. In particular, the high grade serous and high grade endometrioid were evenly integrated, which is consistent with the previously observed similarity of these subtypes as assessed using immunohistochemical markers [25] and gene expression profiles [26].

**Figure 1:**
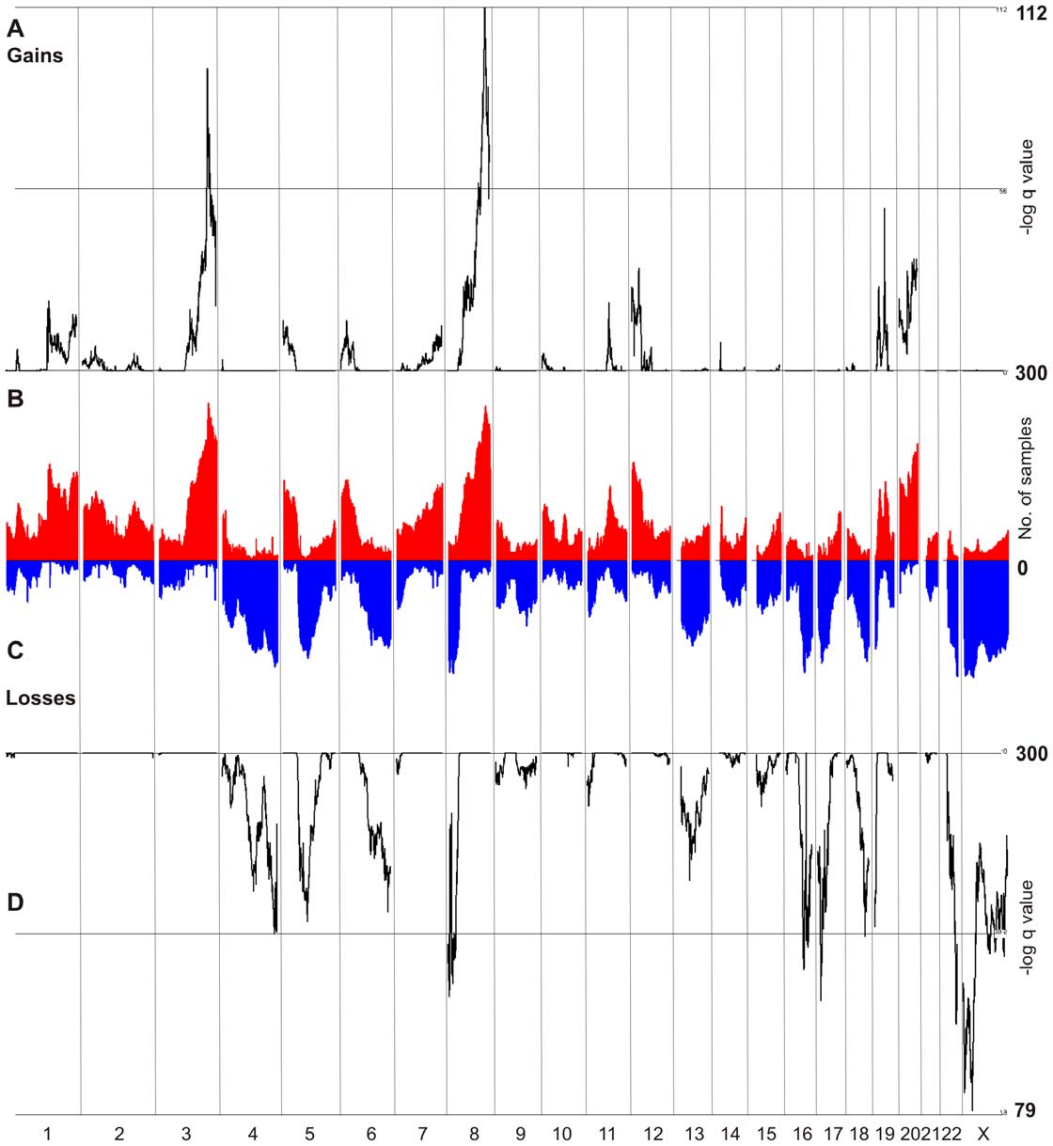
CNA in ovarian cancer. Gains (A) and losses (D) in 240 samples on SNP6 arrays analysed by GISTIC. Gains (B) and losses (C) in 398 samples on various array platforms. Sample segments were overlapped in Partek Genomics Suite v 6.4, creating a data point for each segment defined by copy number breakpoints, and then plotted by sample number.

In order to identify the most relevant CNAs we performed a number of complementary analyses as each method used has strengths and weaknesses that may be complemented by the other. Firstly, GISTIC was applied to all 240 SNP6 samples to identify “focal” and “broad” peaks (as defined in [22]) (Figure 1, Table S2). However, GISTIC cannot readily integrate samples from different platforms. We therefore elected to use a second complementary method to GISTIC: an overall frequency approach that would integrate segmented copy number data independent of platform for analysing our entire 398 sample cohort. As expected, the most significant regions of copy number gain predicted by both GISTIC and overall frequency were located on 3q (63% of samples with CN gain) and 8q (62% samples with CN gain) (Figure 1). Other frequent gains were observed on 20q (47%) and 12p (39%). The most frequent regions of loss identified in this study (chromosomes X, 8p, 22q, 17, 4q, 19p and 16, >40%) are consistent with previous studies by us [15] and others [10], [27]. To select the most relevant genes, we firstly report those in regions of gain and loss with at least 30% frequency or in GISTIC peaks and then identified genes that were also targeted by higher amplitude events even if this was at a lower frequency (Table S2). Since there is no clear consensus on what constitutes a “high-level” amplification, we report regions with frequent gains at log_2_ ratios of >0.6 (in 40 or more samples,10%+), >0.8 (5%+) and >1 (2.5%+). For losses, we considered homozygous deletions (log_2_ ratios of <−1) present in at least 4 samples. The list of genes was prioritised taking into account the frequency of high-amplitude CNA and the overlap with GISTIC (Tables 2 and 3). Specific regions of gain are shown in Figures S2, S3, S4, S5, S6, and S7.

**Table 2:** Selected genes in frequent regions of copy number loss.

Cytoband	Genes	HD^1^	Losses^2^	Region^3^		GISTIC peak^4^		GISTIC broad^5^	
4q35	TRIML1, TRIML2, ZFP42	2	162	179.31*	190.08	189.08	189.50	55.26	191.26
5q11	GPBP1	4	119	56.85	57.99	56.5	56.59	49.48	133.51
5q14	EDIL3	0	154	58.26	94.77	84.07	84.13	49.48	133.51
6q26	PARK2	3	140	139.98	170.69	162.8	162.88	63.13	170.89
8p23	CSMD1	16	178	2.34	6.00	4.22	4.25	0	38.41
10q23	PTEN	8	47	NA	NA	89.71	89.72	NA	NA
13q	RB1, LPAR6, RCBTB2	7	122	46.652	49.76	47.89	48.01	0	114.13
16q21	CDH8	4	176	58.13*	64.01	61.56	62.0	NA	NA
16q23	WWOX	20	153	56.76	82.59	77.56	77.58	69.76	88.81
17p12	MAP2K4, mir-744	6	164	11.26*	12.11	11.9	12.0	0	56.04
17q12	NF1	5	137	24.86	36.68	28.83	28.83	0	56.04
17q12	ACCN	2	140	24.86	36.68	28.83	28.83	0	56.04
18q22	DOK6, CD226, RTTN, SOCS6, CBLN2, NETO1, TMX3, CCDC102B	2–4	159	55.68	75.95	64.9	64.91	20.47	76.12
19p13	FAM148C, SHC2, mir-1302-2	2	141	0.34	4.84	0	0.26	NA	NA
22q13.33	BRD1	5	184	43.77*	49.02	47.89	48.06	15.04	49.58
Xp22.31	NLGN4X, HDHD1A, STS, VCX3A	21–22	183	1.83*	42.77	5.63	5.66	0	154.91
Xp21.1	DMD, mir548f-5	23–26	187	1.83*	42.77	32.68	32.71	0	154.91
Xq21.31	KLHL4, mir-361, DACH2	14–16	160	86.64*	86.89	NA	NA	0	154.91
Xq27.3	SLITRK2, CXorf1, mir-888, mir-890, mir-891, mir-892	10	141	61.65	152.36	144.28	144.75	0	154.91

1 HD, number of homozygous deletions affecting genes shown.

2 Maximum number of losses affecting genes shown.

3 Region (in Mb) with frequency of loss of at least 30% loss unless:

*, >40%, NA  =  not applicable, i.e. no region of frequency >30%.

4 Nearest GISTIC peak if within 5 Mb (otherwise NA).

5 GISTIC broad region containing gene(s), if none then NA.

**Table 3:** Selected genes in frequent regions of copy number gain.

Cytoband	Genes	>1^1^	>0.8^1^	>0.6^1^	>0.3^1^	Amp Region^2^		Gain region^3^		GISTIC peak^4^		GISTIC broad^5^	
1p34.2	BMP8A HEYL LOC728448 MACF1 PABPC4 SNORA55	12	18	32	92	39.22	43.95	NA		39.71	39.89	NA	
1q21.1	ACP6 BCL9 GJA5	10	18	32	146	145.54	145.82	143.95	156.56	145.42	145.67	142.72	247.19
1q21.2	CTSS GOLPH3L HORMAD1	6	12	29	154	NA		143.95	156.56	148.93	148.97	142.72	247.19
3q26.2	MECOM	19	50	105	251	169.39	171.81	167.80	188.04†	170.89	170.92	90.59	199.34
3q29	DLG1 FYTTD1 KIAA0226	5	22	62	192	NA		146.87	198.55*	198.96	198.98	90.59	199.34
5p15.33	EXOC3 CEP72	4	11	34	128	NA		0.26	1.84	0.58	0.67	0	45.87
6p22.3	MBOAT1 ID4	6	12	39	129	NA		17.47	22.19	20.23	20.46	0.32	50.22
6p21.1	PGC TFEB CCND3	10	12	27	85	42.01	42.01	NA		41.78	41.82	0.32	50.22
8q13.2	CSPP1 ARFGEF1 CPA6 PREX2 C8orf34	7	15	42	141	NA		59.81	76.77	69.78	69.88	36.59	146.27
8q22.3	NACAP1 GRHL2 NCALD	4	22	71	180	NA		96.50	143.9*	102.35	102.55	36.59	146.27
8q24.2	MYC PVT1 mir1208	30	73	127	244	125.29	135.41*	117.02	143.9†	129.71	129.75	36.59	146.27
8q24.3	C8orf33 C8orf77	9	25	67	143	NA		84.58	146.27	146.2	146.27	36.59	146.27
11q13.5	C11orf30/EMSY	13	15	33	108	75.46	78.53	NA		77.45	77.57	NA	
11q14.1	RSF1 INTS4 KCTD14 THRSP NDUFC2 ALG8 KCTD21 USP35 GAB2 NARS2	13	20	40	120	75.46	78.53	77.64	77.8	77.45	77.57	NA	
12p13.33-p13.32	ERC1 CACNA1C TSPAN9 EFCAB4B	14	25	51	157	0.73	3.89	0.09	16.41	1.33	1.47	0	36.14
12p12.1	CASC1 KRAS LYRM5	17	26	46	129	15.08	29.45	21.71	27.77	25.21	25.27	0	36.14
19p13.13	STX10 IER2 mir27a CACNA1A CCDC130 ZSWIM4 mir24-2 NANOS3 mir23a RFX1 MRI1 CC2D1A PODNL1 mir181c DCAF15 mir181d	14	27	44	101	12.78	15.27	NA		15.24	15.28	7.45	46.03
19p13.12	EMR3 ZNF333 BRD4 EMR2 SYDE1 ILVBL NOTCH3 EPHX3	12	27	46	114	12.78	15.27	NA		15.24	15.28	7.45	46.03
19q12	CCNE1 C19orf2	36	47	65	126	34.40	35.93*	34.58	35.45	34.997	35.00	7.45	46.03
19q13.2	GMFG LRFN1 PAK4 MED29 PAF1 PLEKHG2 SAMD4B ZFP36 IL29 NCCRP1 SYCN IL28B IL28A	11	20	33	82	44.31	44.81	NA		44.482	44.61	7.45	46.03
20p13	DEFB127 DEFB128 DEFB129	5	20	40	132	NA		0.096	3.53	0.09	0.10	0	62.39
20q11.21	DUSP15 BCL2L1 FOXS1 MYLK2 TPX2	7	23	47	142	NA		29.310	35.02	29.86	29.92	0	62.39
20q13.2	ZNF217 SUMO1P1 BCAS1CYP24A1 PFDN4	9	22	54	172	NA		49.999	62.34*	52.14	52.24	0	62.39
20q13.33	PTK6 GMEB2 EEF1A2	4	17	59	187	NA		49.999	62.34*	61.72	61.82	0	62.39

1 Maximum number of samples with gains of amplitude indicated (log_2_ ratio) at genes shown; not all genes shown will be gained at the maximum frequency.

2 Region (in Mb) with frequency of at least 2.5% high amplitude (>1 log_2_ ratio) gain unless:

*, 5%; NA  =  not applicable, i.e. no region of frequency >2.5%.

3 Region with frequency of at least 30% gain (log_2_ ratio >0.3) unless:

*, >40%;

† , >50%; NA  =  not applicable, i.e. no region of frequency >30%.

4 Nearest GISTIC peak if within 5 Mb (otherwise NA).

5 GISTIC broad region containing gene(s), if none then NA.

In using this flexible approach we found that some regions were only clearly identified by one or the other method. By including a range of higher amplitude CN thresholds and the peaks predicted by GISTIC, additional regions were identified such as gains on chromosomes 1, 6p, 11q, 19 and losses on 5q, 6q26, 10q23, 13q and 18q22. In addition, on high resolution platforms such as the SNP6 array, GISTIC tended to identify very small regions, potentially missing relevant genes. For example, on 3q26 there were two closely spaced peaks of significance in the GISTIC profile (Figure S2). The highest of these, by a very narrow margin (–log q value 93.88 *vs*. 93.43), does not intersect with any genes, while the other peak overlaps with *MECOM* (*MDS/EVI1*); there is good evidence for this gene being an oncogene in ovarian cancer [28]. Thus, relying on GISTIC alone would annotate the 3q26 region as having no genes of interest. In contrast, using a frequency approach, the maximum frequency at all copy number thresholds encompasses *MECOM*.

Similarly, there were other regions for which using a frequency approach missed genes or gave conflicting data. For example, on 19q12, each copy number threshold identified a slightly different region of peak frequency, variously identifying *CCNE1*, *C19ORF2* or no gene in the peak (Figure S3). In contrast, the ability of GISTIC to integrate the amplitude of gain across all samples clearly identified *CCNE1* as the gene in the peak. There is good evidence that *CCNE1* is the correct call since Cyclin E is a key cell cycle protein and its amplification and over expression has been previously identified as a key driver of patient response to chemotherapy in serous ovarian carcinoma [14]. Major conclusions arising from our analysis of individual deletions and amplicons, including insights into potential driver genes, are provided in the Discussion.

### Associations between CN alterations

The concept of cooperative and mutually exclusive genetic alterations has rarely been examined at the level of CNAs or on a genome-wide scale. We wished to know whether there are any CNAs that cooperate in ovarian tumorigenesis, or that are functionally redundant to each other, for example if they act in the same pathway. To measure this we assessed if there were any CNAs that were more or less likely to be associated with each other, more than by chance, using a statistical analysis. Briefly, we counted the number of samples positive for CNA (e.g. a gain) at region A alone, region B alone, both regions and neither region, and compared the findings to the expected co-occurrence based on the total frequency of CNA at A multiplied by the frequency of B. For example, for a frequency of gain at 20q11 of 68/183 (37%) and at 19q12 of 50/183 (33%), we would expect 12% of samples to have both gains. However, we observe an actual frequency of samples with both alterations that is significantly different from this, i.e. 35/183 (19%, p<0.0001), indicating an increase in co-occurrence above the level of chance and thus possibly cooperating CNAs. The method can also be equally used to detect decreases in co-occurrence. When applying this method genome-wide, we applied a multiple testing correction with a FDR of <5%.

**Figure 2:**
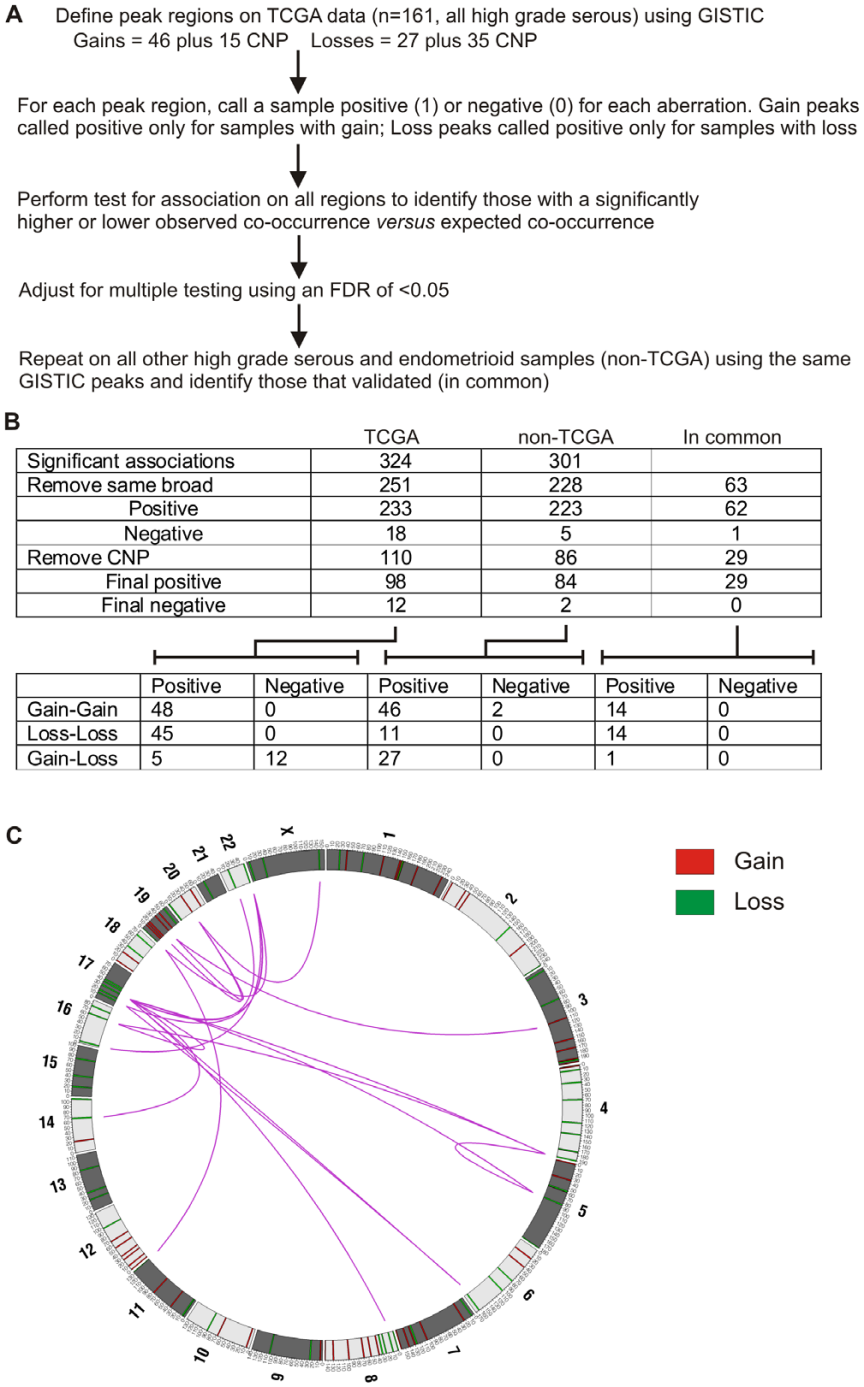
Analysis of association between copy number aberrations. (A) Process for identifying associated aberrations (more detail in Methods S1). (B) Summary of significant associations in each data set and those significant in both. As the table progresses, certain associations are filtered out, with the numbers remaining those that pass the filter. Firstly, associated loci that are within the same broad GISTIC intra-chromosomal region are removed and secondly regions that overlap with a CNP are removed. (C) Circos plot. Outer ring indicates the chromosome position of each aberration (coloured bars). The internal purple lines show the significant inter-chromosomal associations (exclusive of those involving a CNP) that have been validated in the second data set.

We undertook this analysis first using the TCGA data, as it is most homogenous for grade and subtype, and is high resolution. We repeated GISTIC analysis on this data set alone to obtain 46 peaks of copy number gain and 27 of loss (exclusive of regions of normal copy number variation, or copy number polymorphisms (CNPs)). Samples were identified as being positive or negative for each CNA peak, with gain peaks scored as positive for gains only and loss peaks scored as positive for losses only, and an analysis of association was performed as described in the methods. At a false discovery rate of 5%, 305 pairs of regions of aberration were positively correlated and 18 pairs were negatively correlated (Table S3, Figure 2). Some co-occurring GISTIC peaks were located within the same broad GISTIC region and although the GISTIC analysis indicated that these regions of copy number change were distinct, because they are physically closely linked they may not be independent of each other. As independence is necessary for the association test performed, they were not analysed further. We also excluded those associations in which either peak was a CNP, leaving 98 pairs of regions that were positively correlated, all but 16 of which were located on different chromosome arms (Table 4). 12 pairs of regions were negatively correlated.

**Table 4:** List of positively associated CN regions.

	RegionA					RegionB			
Chr	Start^1^	End	Type	Genes^2^	Chr	Start	End	Type	Genes
1	145.42	145.43	Gain	ACP6 BCL9	1	148.94	148.95	Gain	CTSS GOLPH3L HORMAD1
1	145.42	145.43	Gain	ACP6 BCL9	1	151.61	151.62	Gain	S100A12 (S100A8 S100A9)
1	145.42	145.43	Gain	ACP6 BCL9	1	181.84	181.86	Gain	APOBEC4 ARPC5 RGL1
1	145.42	145.43	Gain	ACP6 BCL9	1	233.41	233.45	Gain	ARID4B GGPS1
3	109.186	109.19	Gain	*None*	19	35.15	35.17	Gain	C19orf2
4	190.1	190.23	Loss	*None*	16	61.56	61.58	Loss	*None*
4	190.1	190.23	Loss	*None*	17	28.829	28.834	Loss	ACCN1
5	84.07	84.13	Loss	*None*	17	28.829	28.834	Loss	ACCN1
6	20.325	20.42	Gain	MBOAT1	6	41.78	41.81	Gain	PGC TFEB
6	163.4	163.45	Loss	PACRG	17	11.85	11.88	Loss	DNAH9 MAP2K4 ZNF18 mir744
6	163.4	163.45	Loss	PACRG	17	28.829	28.834	Loss	ACCN1
7	104.33	104.38	Gain	LHFPL3 LOC723809	7	130.22	130.23	Gain	FLJ43663 mir29a mir29b
7	104.33	104.38	Gain	LHFPL3 LOC723809	7	158.17	158.17	Gain	NCAPG2
8	11.07	11.09	Loss	C8orf74 MSRA PINX1 RP1L1 SOX7 UNQ9391 XKR6 mir1322 mir598	17	28.829	28.834	Loss	ACCN1
11	134.43	134.43	Loss	*None*	19	0	0.303	Loss	mir1302-2 FAM138A FAM138C FAM138F FLJ45445 KIR2DL2 KIR2DL4 KIR2DL5A KIR2DL5B KIR2DS1 THEG KIR2DS2 MIER2 KIR2DS3 KIR2DS5 KIR3DP1 KIR3DS1 OR4F17 PPAP2C
12	25.22	25.24	Gain	CASC1 KRAS LYRM5	12	68.16	68.19	Gain	FRS2
12	53.08	53.15	Gain	GTSF1 ITGA5 NCKAP1L ZNF385A	12	68.16	68.19	Gain	FRS2
14	67.37	67.38	Loss	RAD51L1 ZFYVE26	17	28.829	28.834	Loss	ACCN1
16	3.73	3.81	Loss	BTBD12 CLUAP1 CREBBP DNASE1 NLRC3 TRAP1	22	48.58	48.62	Loss	BRD1 C22orf34 LOC90834 (ZBED4)
16	61.56	61.58	Loss	*None*	17	28.829	28.834	Loss	ACCN1
17	11.85	11.88	Loss	DNAH9 MAP2K4 ZNF18 mir744	22	48.58	48.62	Loss	BRD1 C22orf34 LOC90834 (ZBED4)
17	28.829	28.834	Loss	ACCN1	22	15.88	16.19	Loss	CECR1 CECR4 CECR5 CECR6 CECR7 GAB4 IL17RA
17	28.829	28.834	Loss	ACCN1	22	48.58	48.62	Loss	BRD1 C22orf34 LOC90834 (ZBED4)
19	0	0.303	Loss	mir1302-2 FAM138A FAM138C FAM138F THEG FLJ45445 KIR2DL2 KIR2DL4 KIR2DL5A KIR2DL5B KIR2DS1 KIR2DS2 KIR2DS3 KIR2DS5 KIR3DP1 KIR3DS1 MIER2 OR4F17 PPAP2C	22	48.58	48.62	Loss	BRD1 C22orf34 LOC90834 (ZBED4)
19	14.81	14.85	Gain	OR7A10 OR7A17 (OR7A5)	19	35.146	35.17	Gain	C19orf2
19	18.98	19.13	Gain	ARMC6 LOC729991 MEF2B SFRS14 SLC25A42 TMEM161A	19	35.146	35.17	Gain	C19orf2
19	18.98	19.13	Gain	ARMC6 LOC729991 MEF2B SFRS14 SLC25A42 TMEM161A	20	29.86	29.91	Gain	DUSP15 FOXS1 MYLK2 (TTLL9 TPX2)
19	35.15	35.17	Gain	C19orf2	20	29.86	29.91	Gain	DUSP15 FOXS1 MYLK2 (TTLL9 TPX2)
20	29.86	29.91	Gain	DUSP15 FOXS1 MYLK2 (TTLL9 TPX2)	X	144.32	144.38	Loss	CXorf1 SLITRK2 (mir890mir888 mir892a mir892b mir891b)

1 Start and End positions of minimal GISTIC peak in Mbp, hg18.

2 Genes from UCSC RefFlat, hg18, Sept 2009 within wider limits of GISTIC peak. Genes in brackets are within 10 kb of these limits.

In order to validate the associations identified using TCGA data, we repeated the association analysis using the same “TCGA GISTIC-defined” regions as above on all other high grade serous and endometrioid samples (n = 183). For this data set, 296 regions were positively correlated and 5 were negatively correlated. Overall, 29 positive associations and no negative were in common between the two datasets (Figure 2). Of these, 14 were associations between two gains, 11 of which were on the same chromosome, and 14 associations were between two losses. None of the loss-loss associations were intra-chromosomal, because all associations of this type were excluded either for being located in the same broad GISTIC region or for being a CNP; indeed, more of the GISTIC peak losses were CNPs (n = 35) compared to the gains (n = 15) likely due to the unmasking effect loss of heterozygosity has on CNP detection in the tumour [29]. There was a single association between a gain and a loss, between an amplicon on 20q11 and loss of Xq. The strongest positive association between gains on different chromosomes was for amplifications on chromosome 19q12 (most likely targeting *CCNE1*) and at 20q11 (five genes). For losses, the strongest common association was between chromosome 4q and chromosome 17. 17q12 loss was the most promiscuous interactor, with 8 common positive associations.

We identified the genes located in or near positively associated peaks and used gene expression data to evaluate whether any of the genes showed correlation between copy number and expression, and if there were correlation at the level of gene expression across regions (Table S4). We found that the strongest associations across regions involved genes gained on 19q12 or 19p13.11, and genes gained on 20q11. Other positive gene expression associations included *CD47* (gained on 3q13.12) with *UQCRFS1* or *POP4* (both gained on 19q12). CD47 was first identified as an ovarian tumour antigen [30], however there is no known functional association with either 19q12 partner.

### Correlation with clinical parameters and outcome

We used the TCGA clinical data to assess the relationship of copy number and patient outcome using a univariate Cox proportional hazards analysis on the GISTIC peaks (Table S5). Gain on 3q29 was associated with overall survival, however, this correlation was not significant after multiple testing correction. Positive CN associations of 17q12/22q losses and 3q13/19q12 gains were each correlated with overall survival but not progression free survival (Table S5).

Specific patterns of copy number change and genetic instability that correlate with patient outcome, including simplex, sawtooth and firestorm, have been described in breast cancer [31]. The patterns of chromosome aberration in ovarian cancer are difficult to categorise into the groups described by Hicks *et al*. as most are a combination of sawtooth and firestorm. Therefore, we defined a number of different measures of genome instability and analysed their correlation with patient outcome using the TCGA data set (Table S5). These measures included: the number of copy number changes i.e. gains, losses, higher level gains (>0.6 log_2_ amplitude) and total number of segments; the percentage of the genome targeted by copy number change (gain, loss and high level gain); and a “Hicks index” as described [31] for gains, losses and both. The samples were divided into quartiles based on each of these indices and tested for association with clinical outcome using a univariate Cox proportional hazards analysis. Of these measures, only the number of higher amplitude gains (p = 0.019) showed a correlation with progression free survival but not overall survival (Figure S8). The percentage of the genome encompassed in higher level gains was not significant (p = 0.88), suggesting that it is not the proportion of DNA amplified but the number of amplification events that is most important.

## Discussion

Aneuploidy and cytogenetic aberrations have long been recognised as cancer hallmarks. In epithelial cancers, copy number alterations have been shown to be drivers of the cancer phenotype through amplification and over expression of oncogenes like *ERBB2* and loss of tumour suppressors such as *CDKN2A*. Ovarian cancer is both heterogeneous and cytogenetically complex making it difficult to decipher the key genomic regions affected by CNA. Previous studies have generally been underpowered with respect to resolution and/or sample number, at most comprising around 100 cases [10], [11], [12]. This study brings together a large collection of ovarian carcinomas profiled for copy number, which we have analysed using both GISTIC and frequency approaches to provide a definitive annotation of driver alterations. Key regions are summarised in Tables 2 and 3 while a more comprehensive catalogue, encompassing the union of both methods is given in Table S2. Because of the large numbers of genes and regions involved, it is not possible to address all in detail, however the regions mentioned below illustrate some of the insights derived from working with this large data set.

We elected to use complementary analytical approaches as each technique has its own strengths and weaknesses: a frequency approach for regions such as 3q26 was better able to identify the likely driver gene, *MECOM*, whereas for 19q12 the ability of GISTIC to integrate the magnitude of copy number gain for each sample identified *CCNE1*. Using a tiered frequency approach in concert with GISTIC provided a greater depth of understanding in complex regions for which there is no clear driver. Previous studies have identified an amplification on chromosome 11 in 18% of ovarian cancers, and have proposed that the target gene of this event is *EMSY* (*C11ORF30*) [32]. In other cancer types, such as breast cancer, the peak amplification in this region may be different, targeting *EMSY* and/or *CCND1* [33], [34]. In the data presented here, the main amplicon does not appear to be targeting *CCND1*, which is >5 Mb outside the peak region (Figure S4). GISTIC identifies a peak encompassing four genes (*THRSP*, *NDUFC2*, *ALG8* and *KCTD21*), amplification of which have been shown in breast cancer to correlate with over-expression and poor survival [35]. The most frequently targeted gene by low-level gain is *GAB2* (30%). At higher amplitude levels, the amplicon stretches from *RSF1* to *NARS2*, encompassing 11 genes, not including *EMSY* or *PAK1*. However, *EMSY* appears to be targeted in a small proportion of samples (n = 13) by very high level amplification (log_2_>1), including in two samples that are not highly amplified at the more distal site. Similarly, there are two samples that target *CCND1* with very high level amplification that do not cover either more distal region, however the overall frequency of *CCND1* high level amplification is very small (n = 6 with log_2_ >1, 1.5%). The analysis undertaken here has thus found that this apparently simple amplification is in fact more complex, with 3 potentially overlapping targets.

There are some regions for which neither approach was able to identify the “obvious” oncogene. Surprisingly, on 8q24 both the GISTIC and frequency peaks were located ∼500 kb distally to the two previously implicated oncogenes in ovarian cancer within this locus, *MYC* and *PVT1* [36] (Figure S5). This region is distinct from the locus proximal to *MYC* on 8q24 that holds alleles conferring an increased predisposition to cancer [37]. There are no genes in the distal peak region, so the reason for its high frequency of amplification is unclear. From UCSC genome information, this region does appear to contain many repeat and structural variation elements, which could potentially explain the peak shift as an artefact of the array measurement [29], [38]. It is also possible that this region may harbour a long range enhancer for *MYC* and/or *PVT1* or there may be an uncharacterised genetic element in this region that has an additional independent oncogenic effect to that of *MYC* or *PVT1*. This peak feature has not been previously appreciated in the somatic genetics of ovarian cancer, and its detection is a consequence of having a large number of samples at high resolution copy number coverage. Recent sequencing analysis of cancer cell lines has found that amplicons can have a highly complex structure at the base-pair level [39]. It is possible that apparently distinct amplicons may have a common origin with subsequent deletion and rearrangement of internal material.

The strength of this study in having a large number of samples provides numerous additional insights to copy number features of ovarian cancer. Firstly, a number of studies in other cancer types have found that low frequency events present in only a few percent of cases may still be very important for the genesis of the case in which they are found, whether these are point mutations [40] or copy number changes [41]. These types of events are difficult to detect without a large number of samples. Lower frequency but high-amplitude events that may be of relevance to ovarian cancer were readily detected here. The most notable of these as having the highest proportion of high-level amplicons (34/126 of samples with a gain had a log_2_ value of >1) was on 19q12, targeting *CCNE1*. The next highest was also on chromosome 19, at 19p13.1, with two apparent peaks (Figure S3). The peak identified by GISTIC contained the gene *BRD4* and was the highest by low-level gain frequency. *BRD4* appears a likely target, as it is involved in an oncogenic translocation in midline carcinoma [42]. However, at higher amplitude gains, the frequency peak shifts more distal, for example the gene *CACNA1A* was targeted by the most number of very highly amplified samples on 19p (n = 14 at log_2_ >1). Again this illustrates why using a single method of gene prioritisation may miss potential oncogenes. GISTIC alone would have missed the second peak that may be important for a low frequency of high-amplitude gain, whereas a peak frequency approach using a single cut-off would also miss potentially important genes. Of course, neither method can unequivocally prove a role for any of the identified genes in ovarian cancer but do provide a rational basis on which to undertake comprehensive functional or nucleotide level mutation analysis.

With a large number of samples we were able to refine regions of gain on other chromosomes. For example, 20q has long been recognised as having a high frequency of gain, yet the proposed target gene(s) vary from study to study. This variability may be a stochastic consequence of low sample numbers in each study, or additionally because there are multiple genes each involved in only a sub-set of cancers or each with a mild oncogenic effect. Our data would support the view that there are multiple drivers at 20q. The most reproducible of the peaks observed between different thresholds and analyses were at approximately 29–30 Mb (20q11.21), 52 Mb (20q13.2) and 62 Mb (20q13.33) (Figure S6). Many samples (166/398) co-amplified more than one region, however, 5.3–6.5% of samples amplified only one of each of the above three regions. The 20q regions encompass previously noted genes including *TPX2*, *BCAS1*, *ZNF217* and *PTK6*. Interestingly, some previously characterised oncogenes such as *AURKA* [43] were not in peak regions or frequently amplified at a high level.

On chromosome 12p, both GISTIC and the peak frequency of high level amplification clearly identified *KRAS* as the target for some samples (Figure S6). Interestingly, although activating point mutations in *KRAS* in ovarian cancer are observed predominantly in low grade serous subtype and rarely in high grade cases, of the 25 samples with an amplification at *KRAS* of at least 0.8 (log_2_) in amplitude, all but one were high-grade serous, suggesting an alternative mechanism for activation of this pathway in a subset of high grade serous carcinomas. Elsewhere on 12p, the main frequency peak for low-level gain was located at the distal end, for which oncogenes have not been previously characterised but may include *ERC1*, *EFCAB4B* and *TSPAN9*. *ERC1* is a translocation partner of *RET* in AML [44].

Chromosome 1 appears to be targeted by several amplifications (Figure S7). There are two amplicons within 5 Mb of each other on 1q, which appear quite distinct, and yet share 85% of samples at a >0.3 (log_2_) level and 100% at an amplitude threshold of log_2_ ratio>1. It is possible that the apparent gap between these amplicons may be artefactual and a consequence of poor genome mapping and highly repetitive sequence in this region close to the centromere. There are several interesting genes within the amplicons, including *BCL9* (*B-cell CLL/lymphoma 9*), which has recently been characterised as an oncogene in colon carcinoma and multiple myeloma, with a role in the Wnt pathway [45]. Chromosome 1p is particularly interesting, as the amplicon in this region has not been previously noted in ovarian cancer, most likely because it is a highly focal, low frequency event (3% at log_2_ >1, 23% at log_2_ >0.3) that would likely only be detected with a large dataset of high-resolution arrays. Genes within this region include *HEYL*, *BMP8B* and *MYCL1*. *HEYL*, (*Hairy/enhancer of split related with YRPN motif-like protein*) is one of a group of transcription factors that act to inhibit gene expression and are thought to be involved in mediating Notch signalling, possibly with a role in epithelial-to-mesenchymal transition [46]. *HEYL* was found to have increased expression associated with amplification in small cell lung cancer [47]. *BMP8B* encodes a bone morphogenic protein, which are signalling peptides in the TGF-beta family [48]. *MYCL1* is a *MYC* oncogene homolog also identified as being amplified and overexpressed in small cell lung cancer [49].

The large data set collected here also enabled us to confirm previous observations that homozygous deletions are common in the genes *CSMD1*, *DMD*, *NF1*, *RB1* and *PTEN* [15], [50]. Interestingly, frequently co-deleted with *DMD* is the microRNA *hsa-mir-548f-5*, a member of a large family of microRNAs predicted to have regulatory functions relevant to cancer [51]. We also note a high frequency of HDs in *WWOX*, and at several regions on chromosome X, including the novel targets *STS* (*Steroid sulfatase*, involved in estrogen synthesis), *KLHL4* (*Kelch-like 4*, with actin binding homology) and *SLITRK2* (*SLIT and NTRK-like family, member 2*, an integral membrane protein). Co-deleted with *SLITRK2* were a group of 6 microRNA genes including *hsa-mir-888* and *hsa-mir-890* among others. None of these microRNAs have been characterised with validated targets.

Although the insights obtained from this data set are highly informative in helping to determine key regions and their likely drivers, the interpretation of biological relevance is restricted to individual gene information, which is often of limited value since it is possible to construe a cancer-related function for most genes. More direct relevance to cancer development may be ascertained through detailed functional analysis in model systems. However, rather than considering genes in isolation, what would be most interesting is to identify the genes that co-operate during tumorigenesis. One way of doing this is to undertake a pathway analysis to link the genes in various copy number aberrations. However, this analysis is generally “noisy” and likely to identify multiple interactions between the many genes affected by copy number change without necessarily picking the key pathways. An alternative method is to locate those CNAs that interact genetically. We first attempted to dissect this using unsupervised hierarchical clustering of copy number data, but were unable to identify separate genetic subgroups or interacting regions, apart from a group with few genetic changes that was biased towards low-grade serous and low-grade endometrioid samples. We undertook a different type of analysis by a novel bioinformatic method that identifies genomic regions that are more or less likely to be associated with each other than would be expected by chance, given their base frequency (Table 4). The method relies on having a large number of samples available, and with a data set as large as assembled here, we were able to perform the analysis on a test set (TCGA SNP6 data) and also a validation set (all other high grade serous and endometrioid samples). This highly stringent analysis identified a number of common positive associations, for example between amplification of *CCNE1* and genes on 20q or 3q. A recent study performed a similar analysis in glioblastoma looking for positive associations only, and identified 21 validated inter-chromosomal interactions with aberration frequencies of >10% [52]. In contrast we identified 29 interactions (18 inter-chromosomal). None of the interactions were in common, however, this is unsurprising given the very different genetic landscapes of the two tumour types, in particular the increased genetic complexity of ovarian carcinomas compared to glioblastomas. In comparison to previous ovarian cancer studies, we did not find previous associations such as 19q/12p gains [7], or any of 13 inter-chromosomal associations in [10]. However, this is also not surprising as the first analysis used low-resolution 10 K SNP array data followed by fluorescence *in situ* hybridisation of selected loci, while the second performed the analysis on somewhat different GISTIC peaks in many fewer samples and used a Pearson correlation with a low-stringency FDR (0.25).

It is notable that there were many more positive associations identified compared with negative associations. A similar study in breast cancer failed to find any significant negative associations [53]. Positive associations are assumed to identify genes whose activity may be in different but complementary pathways. Negative associations are thought to be redundant aberrations in the same pathway (e.g. *BRAF* and *KRAS* mutations). The differences in these association types may influence the process of their selection. To be selected for together above the level of chance, positive associations will act positively together in synergy. Thus, for one to be selected for there is a strong reliance on the other also being present, as each alone will provide little selective advantage, making their detection straightforward. In addition, for some associated CNAs, they may in fact be physically linked through chromosome translocation events and perforce selected together. In contrast, negative associations are acted on individually by selection – each provides a selective advantage when present first of all in the cell, but the eventuality of the second event occurring subsequently will not provide a selective disadvantage, and may be carried along as a passenger event. In the case of point mutations such as in *BRAF* and *KRAS*, the probability of one mutation occurring subsequent to the other is extremely small, so they are seldom seen together. However, for large-scale copy number alterations, the cytogenetic instability of ovarian cancer means it may be inevitable that a second event will occur by chance in some cases and be carried along as a passenger. Thus the power to detect negative associations in the current analysis may be low.

Finally, we used the clinical annotations available with the TCGA data to assess the correlation of various copy number features with survival. We identified a number of correlations, including of the number of higher amplitude gains with worse overall survival, however these were generally weak and did not pass multiple testing. It was somewhat concerning that no CNAs were strongly associated with survival, given that several recent studies have identified associations with poor outcome for amplification at genes such as *CCNE1* [13], [14]. One explanation could be that the TCGA sample set is not selected in order to target specific clinical questions compared with other studies. Another possibility is that within this generally high grade serous ovarian cancer cohort, the overall consistency of the common aberrations may mean that there is little “dynamic range” to detect differences associated with copy number, and that variance in outcome may have more to do with constitutive factors specific to the individual such as their immune system or pharmacogenomics.

### Conclusion

In this study we have brought together a very large cohort of ovarian cancer cases profiled using high-resolution SNP Mapping arrays to identify the genome-wide frequency of copy number aberrations and their interactions. In addition to the well known frequent events, using this large data set has developed our view of the rare but recurrent events contributing to ovarian cancer, consistent with the “mountains and hills” analogy proposed for other epithelial cancer types [54]. We identified a number of co-occurring aberrations that may co-operate in ovarian tumourigenesis, such as gain of 19q with 20q or 3q, gain of 20q with X loss and loss of 17q with multiple partners. The significance of these aberrations and the validation of the genes underlying the copy number changes remains to be assessed through comprehensive functional and nucleotide level sequence analysis.

## Supporting Information

Figure S1 Comparison of samples run on different array platforms. A. Overall frequency plot of gains and losses for SNP6 TCGA (n = 157), SNP6 PMCC (n = 83), 500 K PMCC (n = 27), 250 K Japanese (n = 23) and 50 K PMCC (n = 108). All platforms used the same log2 threshold of ± 0.3. B. Hierarchical clustering of samples. Samples were scored as positive (red) or negative (blue) for gains and losses identified in all SNP6 samples by GISTIC. Sample source, histological subtype and grade are indicated in colour at the top. There is no apparent grouping by array source that would suggest a batch effect of the arrays, apart from the Japanese 250 K samples (grade unknown), which tend to have few alterations and cluster with the low-grade endometrioid samples at the left-hand side of the dendrogram.
(0.06 MB PDF)

Figure S2 Gain on 3q. A. Frequency of gain on 3q is shown at various amplitude thresholds. Note the different scales for each threshold. The GISTIC -log q value is plotted above, as is the extent of the broad GISTIC region identified (red bar). GISTIC peaks are indicated by arrows. B. Zoomed in view of 3q26. Each of the minimal peak frequency regions for each CN amplitude is shown by a coloured box. The GISTIC peak is indicated by the red box. Below are shown the genes from the UCSC genome browser.
(0.05 MB PDF)

Figure S3 Gain on chr19. Frequency of gain on chr19 is shown at various amplitude thresholds. Note the different scales for each threshold. The GISTIC -log q value is plotted above, as is the extent of the broad GISTIC region identified (red bar). GISTIC peaks are indicated by arrows. Zoomed in views of 19p13 (B) and 19q12 (C) Each of the minimal peak frequency regions for each CN amplitude is shown by a coloured box. The GISTIC peak is indicated by the red box. Below are shown the genes from the UCSC genome browser.
(0.07 MB PDF)

Figure S4 Gain on 11q. A. Frequency of gain on 11q is shown at various amplitude thresholds. Note the different scales for each threshold. The GISTIC -log q value is plotted above; GISTIC peaks are indicated by arrows. Asterisks show 3 possible amplicons B. Zoomed in view of 11q13–14. Each of the minimal peak frequency regions for each CN amplitude is shown by a coloured box. The GISTIC peak is indicated by the red box. Below are shown the genes from the UCSC genome browser.
(0.05 MB PDF)

Figure S5 Gain on 8q. A. Frequency of gain on 8q is shown at various amplitude thresholds. Note the different scales for each threshold. The GISTIC -log q value is plotted above, as is the extent of the broad GISTIC region identified (red bar). GISTIC peaks are indicated by arrows. B. Zoomed in view of 8q24. Each of the minimal peak frequency regions for each CN amplitude is shown by a coloured box. The GISTIC peak is indicated by the red box. Below are shown the genes from the UCSC genome browser.
(0.05 MB PDF)

Figure S6 Gains on chr20 and chr12. A. Frequency of gain on chr20 is shown at various amplitude thresholds. Note the different scales for each threshold. The GISTIC -log q value is plotted above, as is the extent of the broad GISTIC region identified (red bar). GISTIC peaks are indicated by arrows. Zoomed in views of 20q11 (B), 20q13.2 (C) and 20q13.33 (D). Each of the minimal peak frequency regions for each CN amplitude is shown by a coloured box. The GISTIC peak is indicated by the red box. Below are shown the genes from the UCSC genome browser. E. Gain on 12p. Frequency of gain on chr12 is shown at various amplitude thresholds. Note the different scales for each threshold. The GISTIC -log q value is plotted above, as is the extent of the broad GISTIC region identified (red bar). GISTIC peaks are indicated by arrows. F. Zoomed in view of 12p, with various genes indicated.
(0.10 MB PDF)

Figure S7 Gain on chr1. A. Frequency of gain on chr1 is shown at various amplitude thresholds. Note the different scales for each threshold. The GISTIC -log q value is plotted above, as is the extent of the broad GISTIC region identified (red bar). GISTIC peaks are indicated by arrows. Zoomed in views of 1p34 (B) and 1q21 (C). Each of the minimal peak frequency regions for each CN amplitude is shown by a coloured box. The GISTIC peak is indicated by the red box. Below are shown the genes from the UCSC genome browser.
(0.07 MB PDF)

Figure S8 Survival analysis. A. Kaplan Meier plot of overall survival with samples divided into quartiles based on the number of gains >0.6 (log2). P = 0.045 after a Cox proportional hazard model analysis. 1, 0–18 segments; 2, 19–36 segments; 3, 37–60 segments; 4, >60 segments. B. Kaplan Meier plot of overall survival with residual macroscopic disease as a factor.
(0.08 MB PDF)

Methods S1 Supplementary methods.
(0.06 MB DOC)

Table S1 Full list of samples.
(0.18 MB XLS)

Table S2 Full list of frequent aberrations and genes.
(0.71 MB XLS)

Table S3 Full list of associated aberrations.
(2.82 MB XLS)

Table S4 Expression of genes within associated aberrations.
(0.04 MB XLS)

Table S5 Clinical correlations of aberrations.
(0.05 MB XLS)
