# Supplementary figures and images for: Copy Number Analysis Identifies Novel Interactions Between Genomic Loci in Ovarian Cancer
Source: PLoS One. 2010 Sep 10;5(9):e11408. doi: 10.1371/journal.pone.0011408 (PMC2937017; doi:10.1371/journal.pone.0011408)

Figure S1

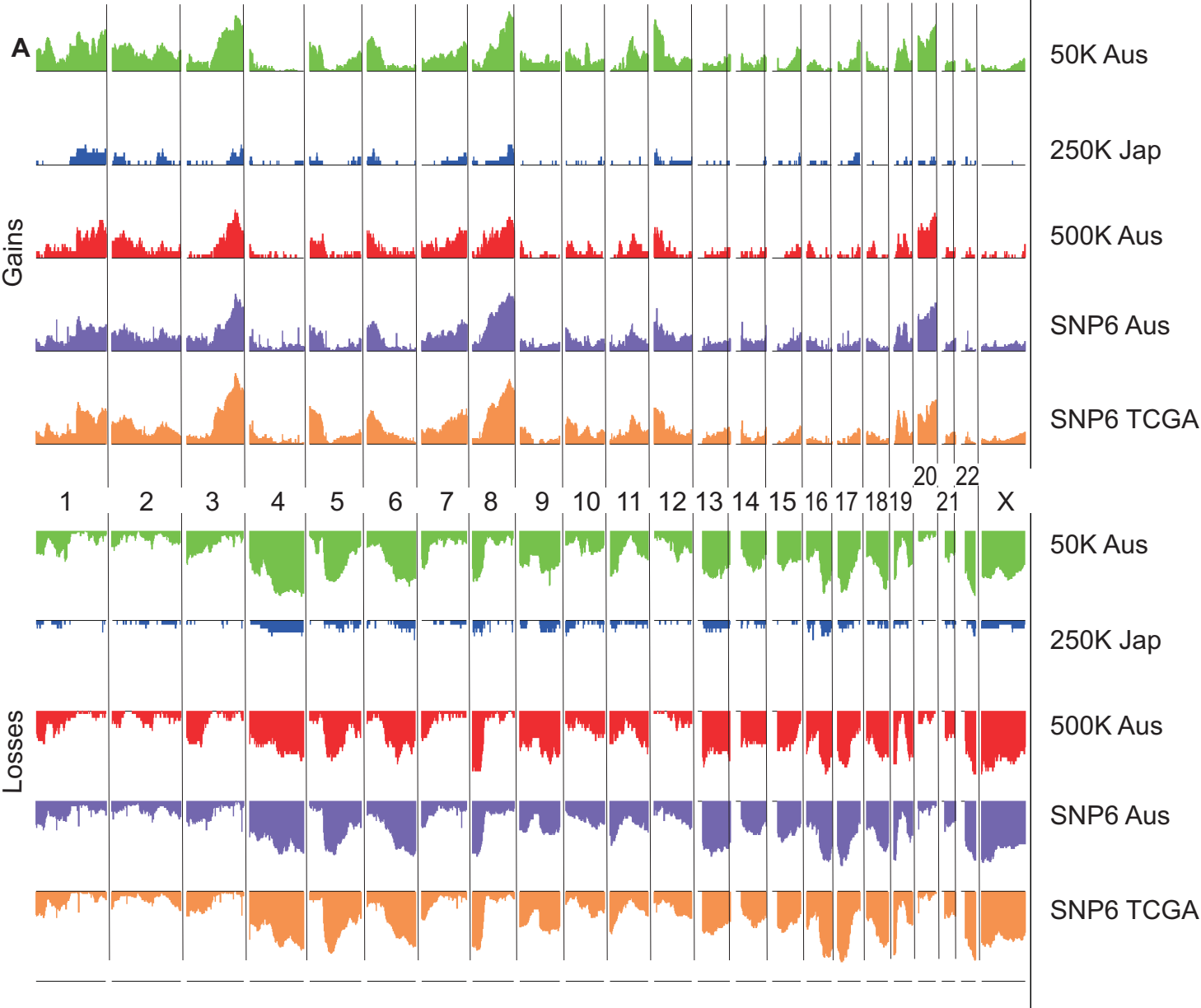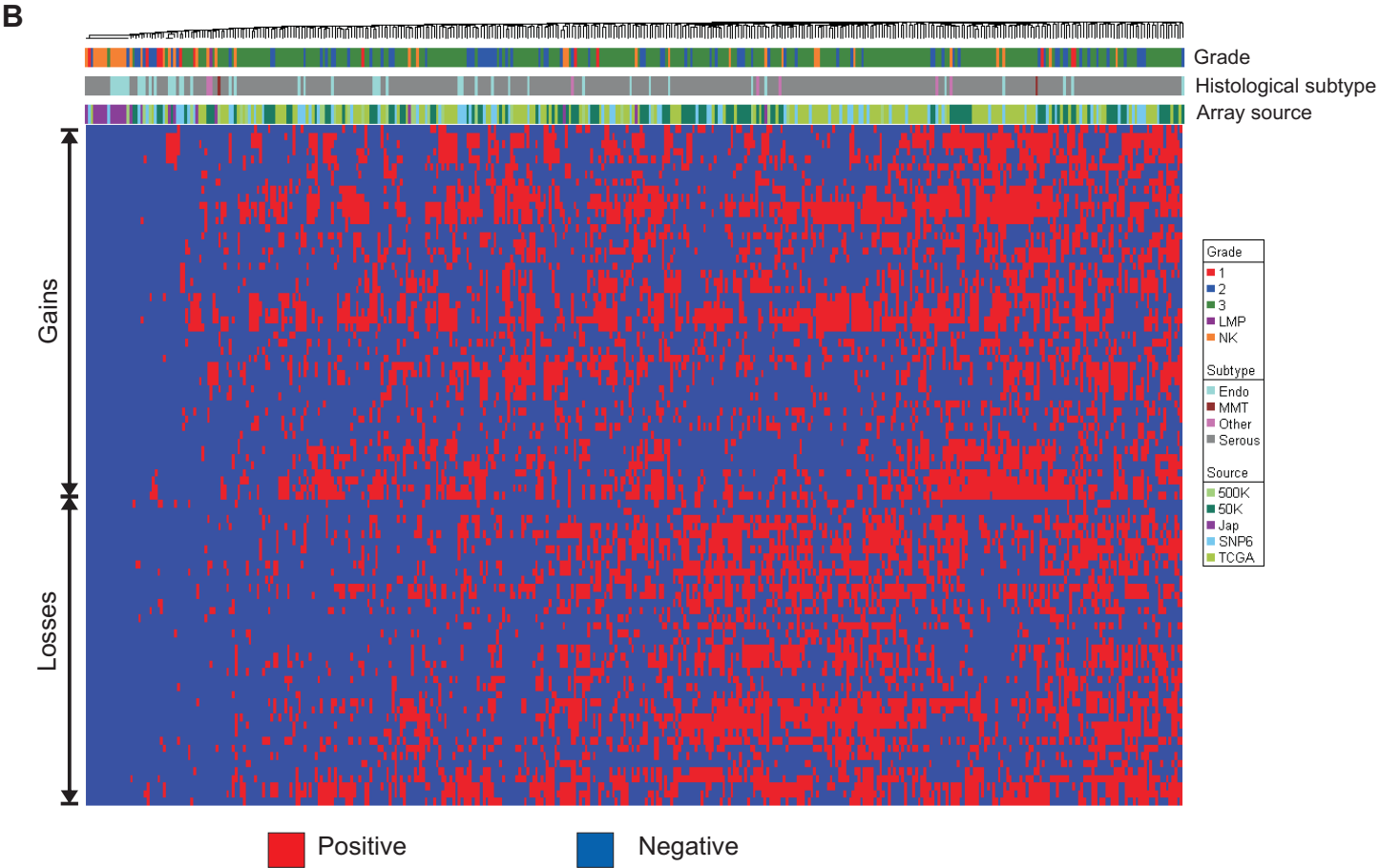

Supplement: Figure S1 — Comparison of samples run on different array platforms. A. Overall frequency plot of gains and losses for SNP6 TCGA (n = 157), SNP6 PMCC (n = 83), 500 K PMCC (n = 27), 250 K Japanese (n = 23) and 50 K PMCC (n = 108). All platforms used the same log2 threshold of ± 0.3. B. Hierarchical clustering of samples. Samples were scored as positive (red) or negative (blue) for gains and losses identified in all SNP6 samples by GISTIC. Sample source, histological subtype and grade are indicated in colour at the top. There is no apparent grouping by array source that would suggest a batch effect of the arrays, apart from the Japanese 250 K samples (grade unknown), which tend to have few alterations and cluster with the low-grade endometrioid samples at the left-hand side of the dendrogram. (0.06 MB PDF) [file pone.0011408.s001.pdf]

Figure S2 - 3q

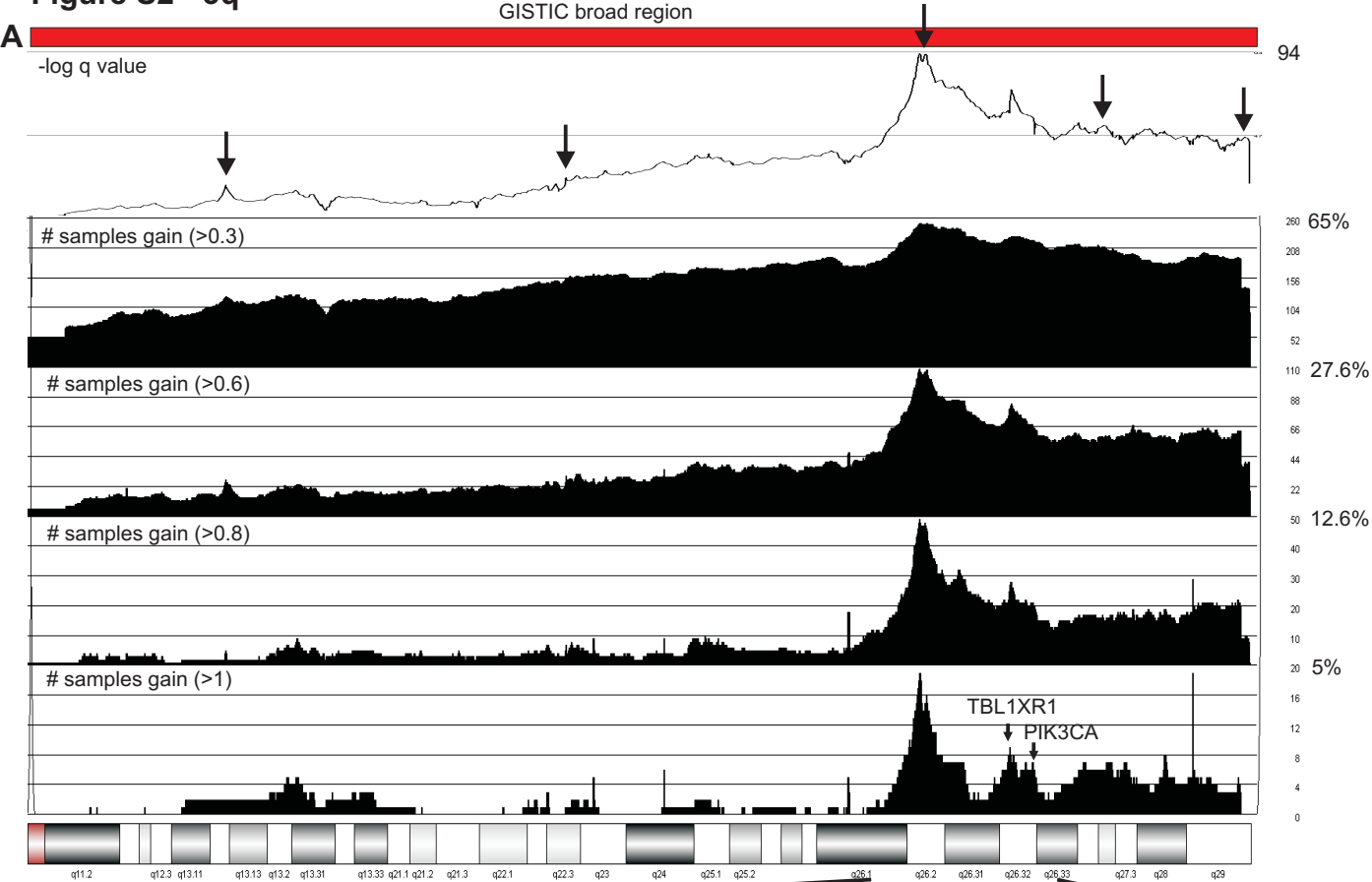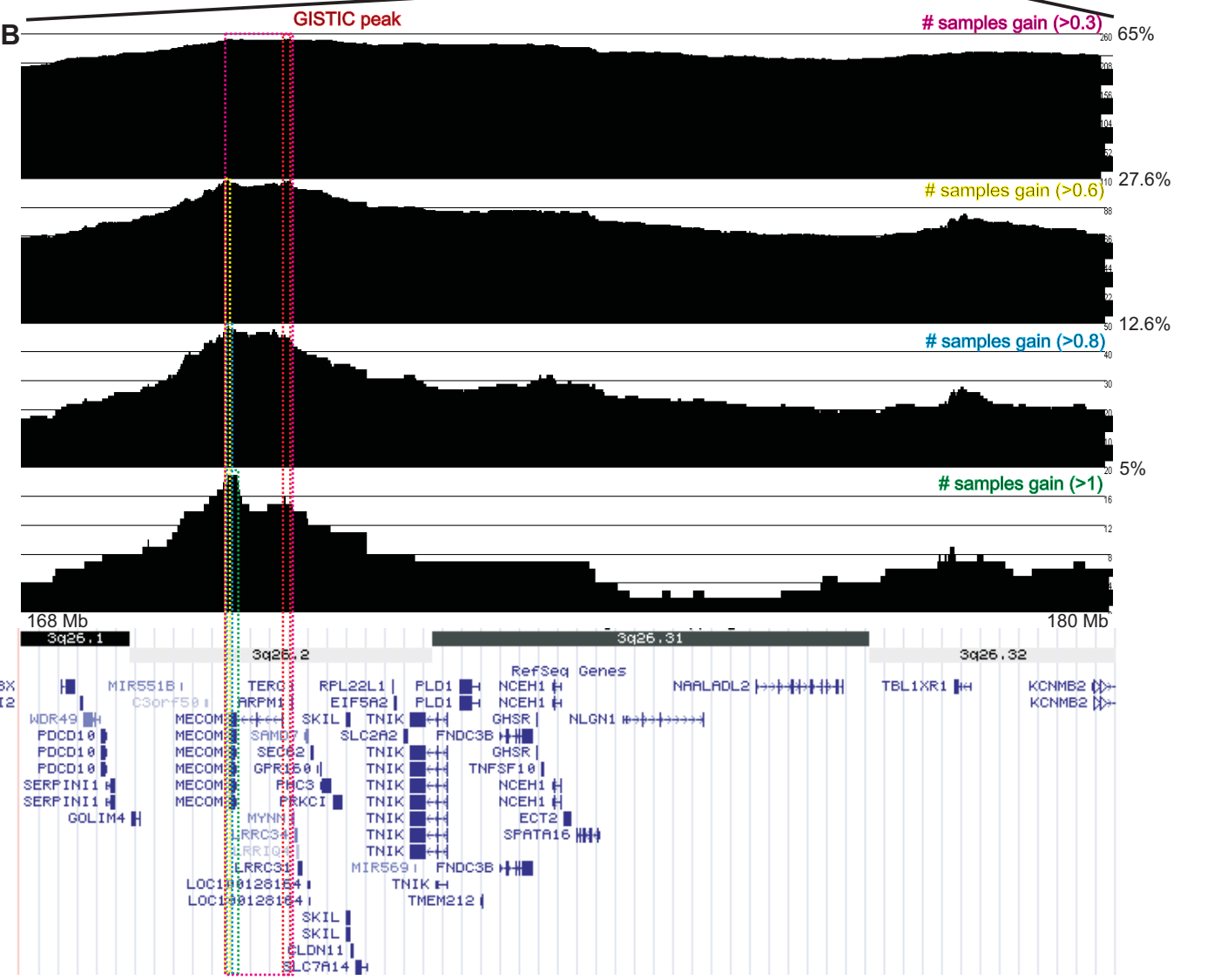

Supplement: Figure S2 — Gain on 3q. A. Frequency of gain on 3q is shown at various amplitude thresholds. Note the different scales for each threshold. The GISTIC -log q value is plotted above, as is the extent of the broad GISTIC region identified (red bar). GISTIC peaks are indicated by arrows. B. Zoomed in view of 3q26. Each of the minimal peak frequency regions for each CN amplitude is shown by a coloured box. The GISTIC peak is indicated by the red box. Below are shown the genes from the UCSC genome browser. (0.05 MB PDF) [file pone.0011408.s002.pdf]

[illegible]

Figure S3 - 19q12

C

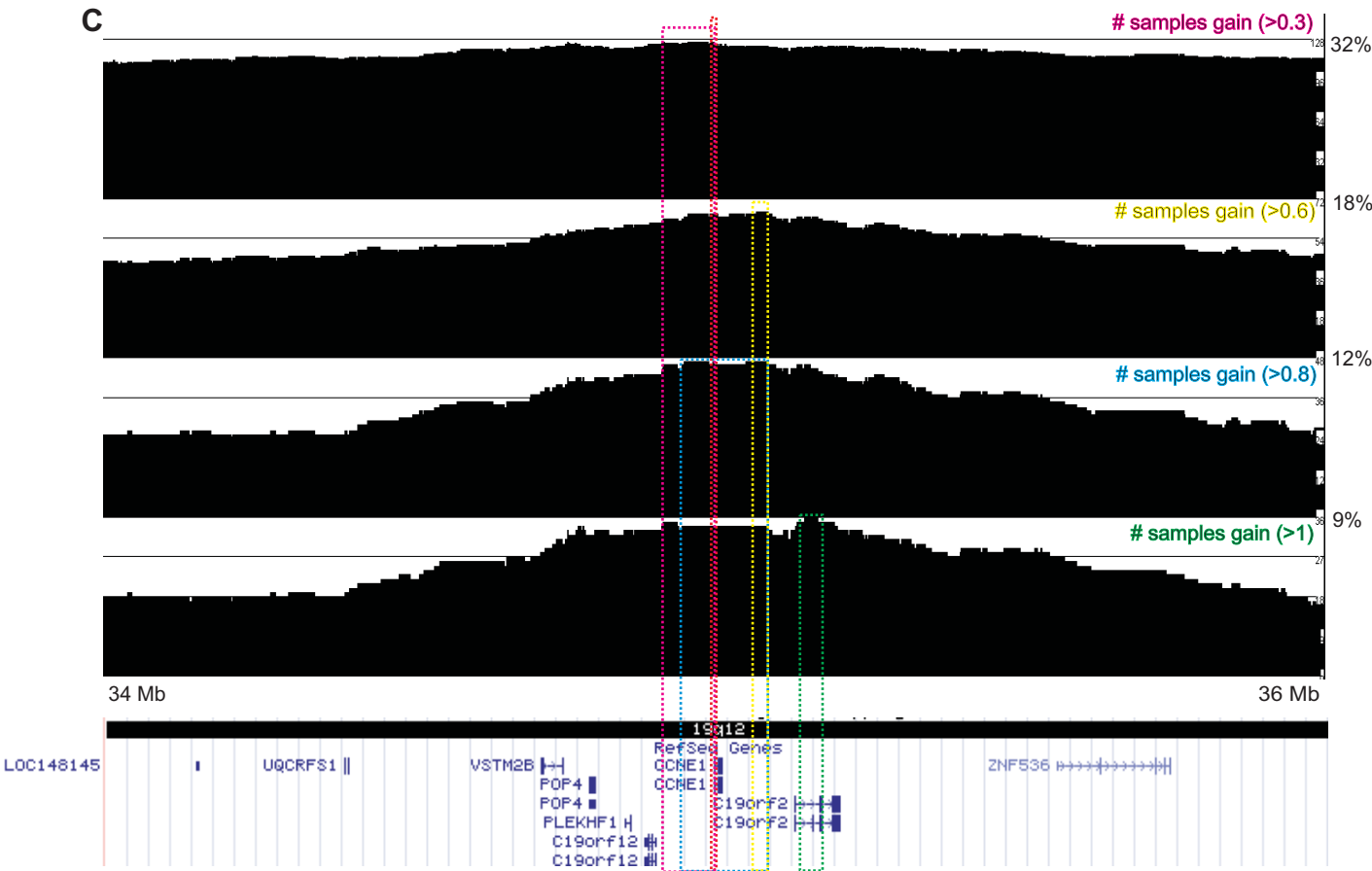

Supplement: Figure S3 — Gain on chr19. Frequency of gain on chr19 is shown at various amplitude thresholds. Note the different scales for each threshold. The GISTIC -log q value is plotted above, as is the extent of the broad GISTIC region identified (red bar). GISTIC peaks are indicated by arrows. Zoomed in views of 19p13 (B) and 19q12 (C) Each of the minimal peak frequency regions for each CN amplitude is shown by a coloured box. The GISTIC peak is indicated by the red box. Below are shown the genes from the UCSC genome browser. (0.07 MB PDF) [file pone.0011408.s003.pdf]

**A**

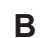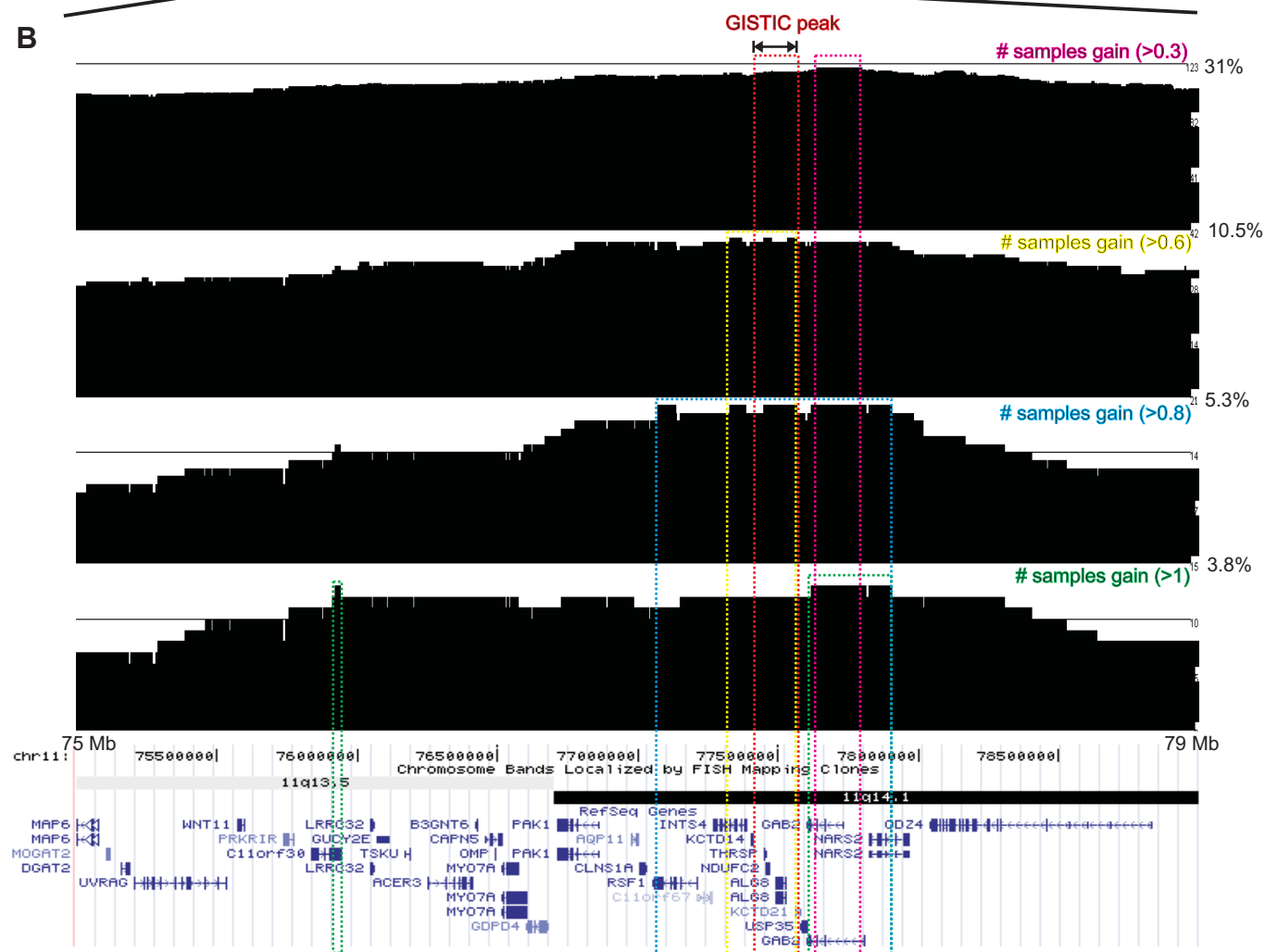

Supplement: Figure S4 — Gain on 11q. A. Frequency of gain on 11q is shown at various amplitude thresholds. Note the different scales for each threshold. The GISTIC -log q value is plotted above; GISTIC peaks are indicated by arrows. Asterisks show 3 possible amplicons B. Zoomed in view of 11q13–14. Each of the minimal peak frequency regions for each CN amplitude is shown by a coloured box. The GISTIC peak is indicated by the red box. Below are shown the genes from the UCSC genome browser. (0.05 MB PDF) [file pone.0011408.s004.pdf]

Figure S5 - 8q

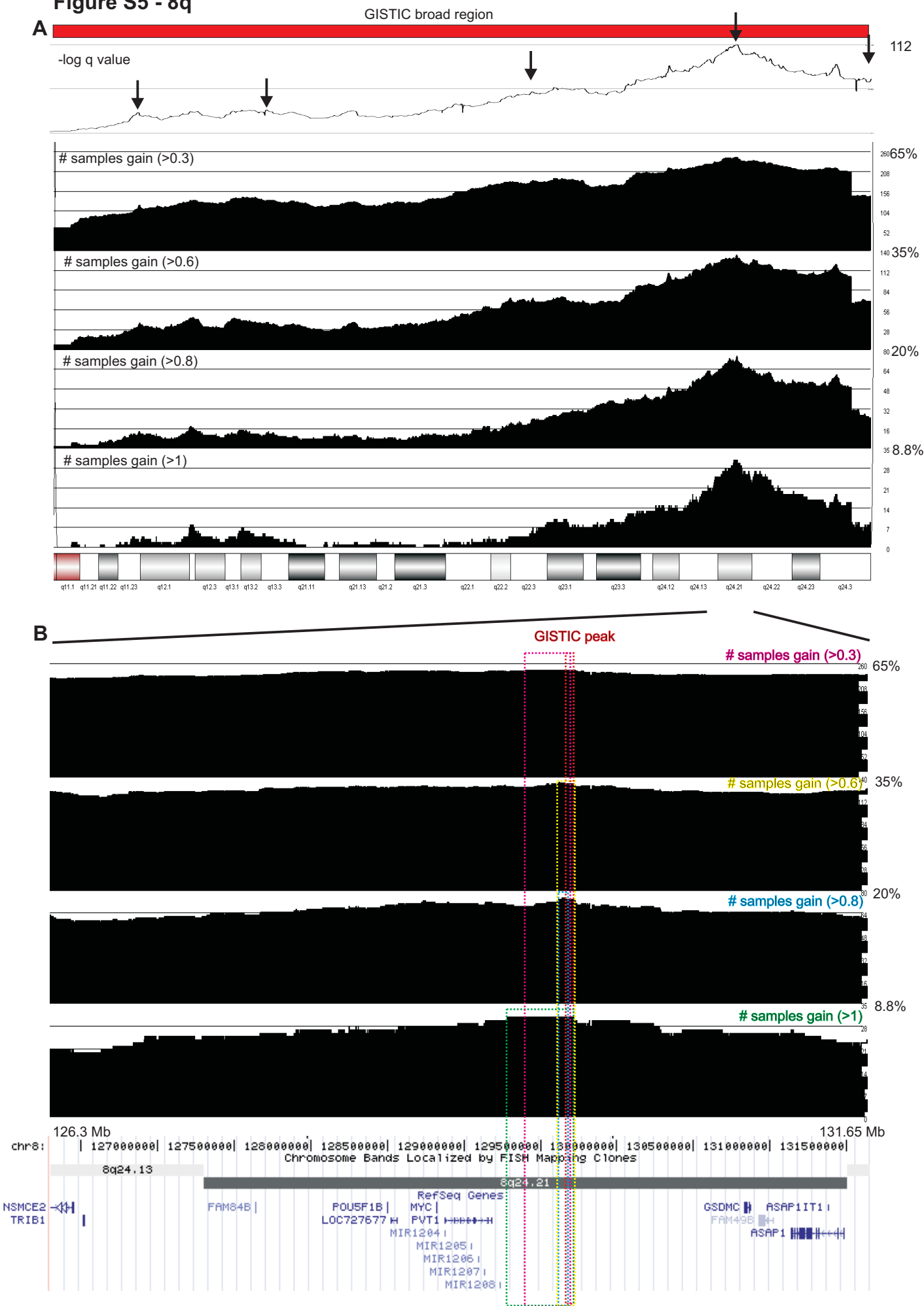

Supplement: Figure S5 — Gain on 8q. A. Frequency of gain on 8q is shown at various amplitude thresholds. Note the different scales for each threshold. The GISTIC -log q value is plotted above, as is the extent of the broad GISTIC region identified (red bar). GISTIC peaks are indicated by arrows. B. Zoomed in view of 8q24. Each of the minimal peak frequency regions for each CN amplitude is shown by a coloured box. The GISTIC peak is indicated by the red box. Below are shown the genes from the UCSC genome browser. (0.05 MB PDF) [file pone.0011408.s005.pdf]

**C**

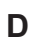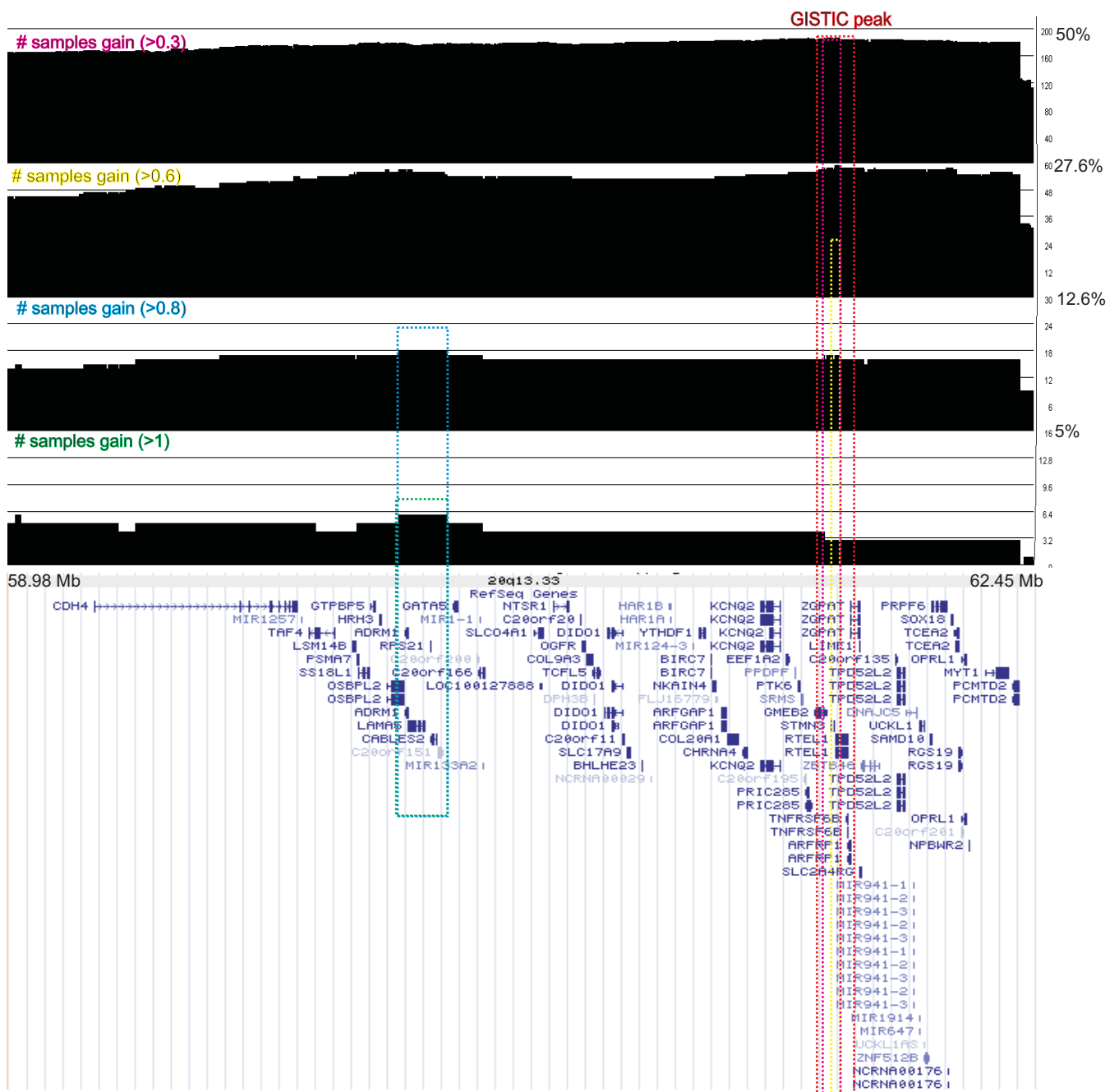

Figure S6 - chr12

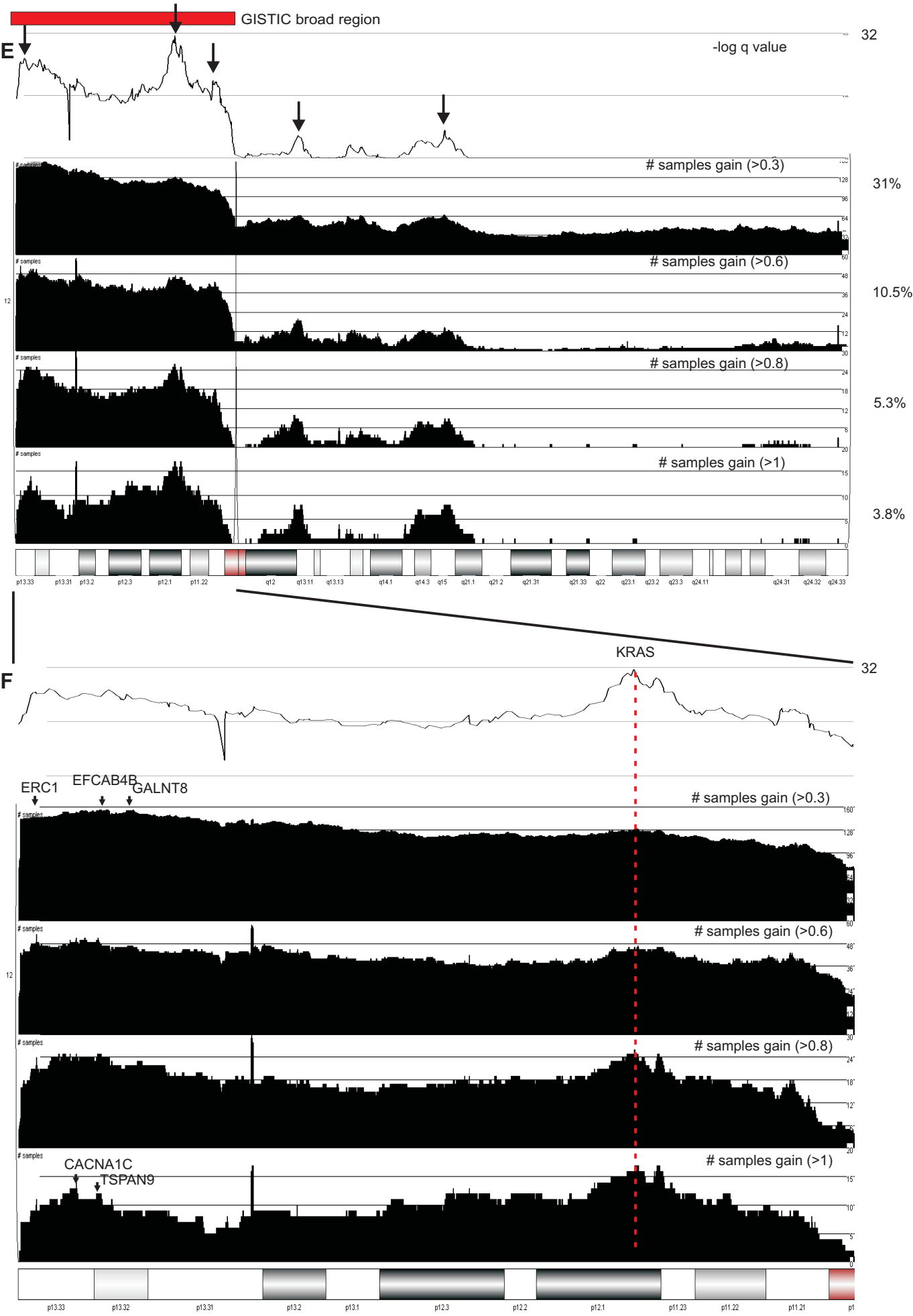

Supplement: Figure S6 — Gains on chr20 and chr12. A. Frequency of gain on chr20 is shown at various amplitude thresholds. Note the different scales for each threshold. The GISTIC -log q value is plotted above, as is the extent of the broad GISTIC region identified (red bar). GISTIC peaks are indicated by arrows. Zoomed in views of 20q11 (B), 20q13.2 (C) and 20q13.33 (D). Each of the minimal peak frequency regions for each CN amplitude is shown by a coloured box. The GISTIC peak is indicated by the red box. Below are shown the genes from the UCSC genome browser. E. Gain on 12p. Frequency of gain on chr12 is shown at various amplitude thresholds. Note the different scales for each threshold. The GISTIC -log q value is plotted above, as is the extent of the broad GISTIC region identified (red bar). GISTIC peaks are indicated by arrows. F. Zoomed in view of 12p, with various genes indicated. (0.10 MB PDF) [file pone.0011408.s006.pdf]

22

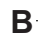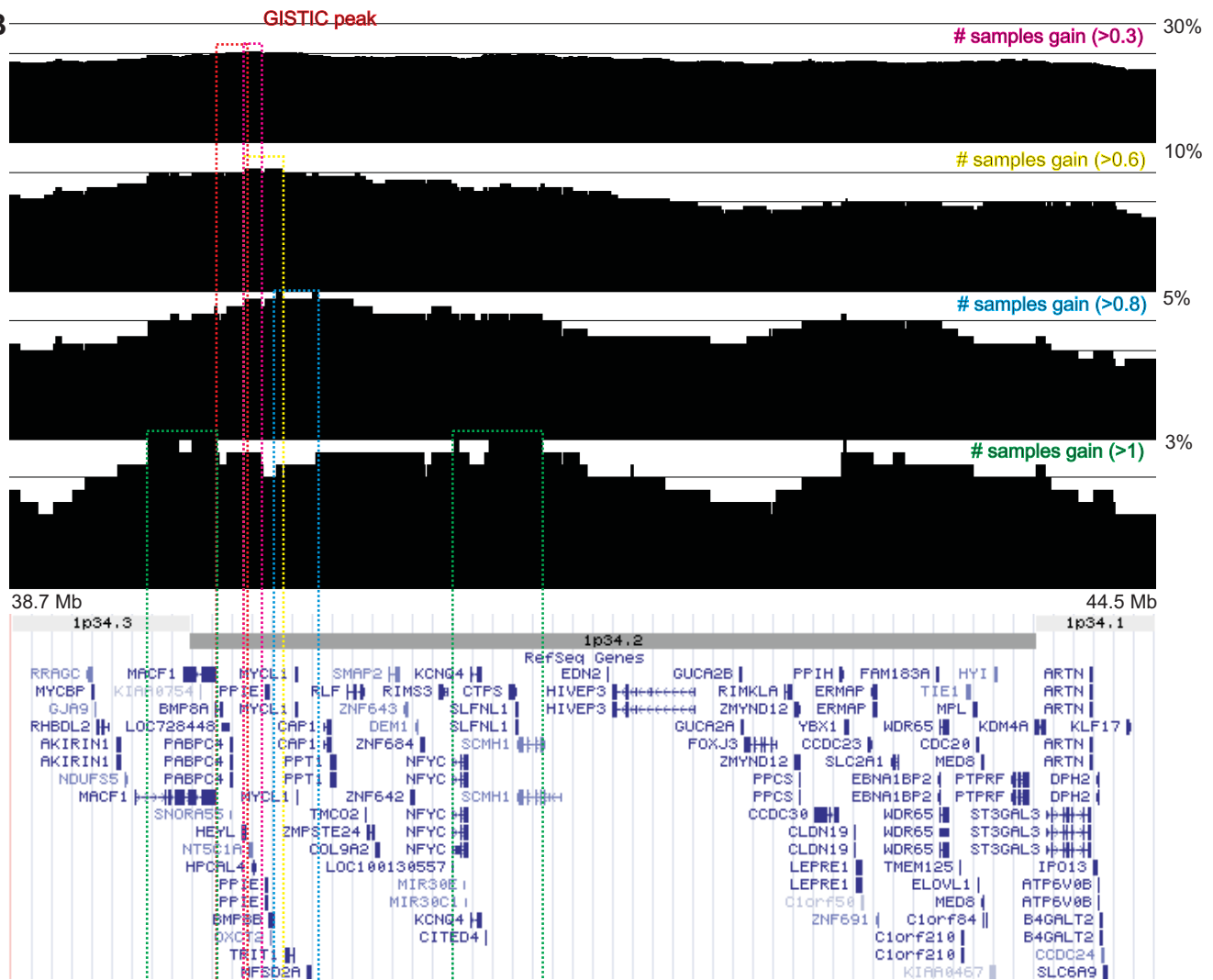

C

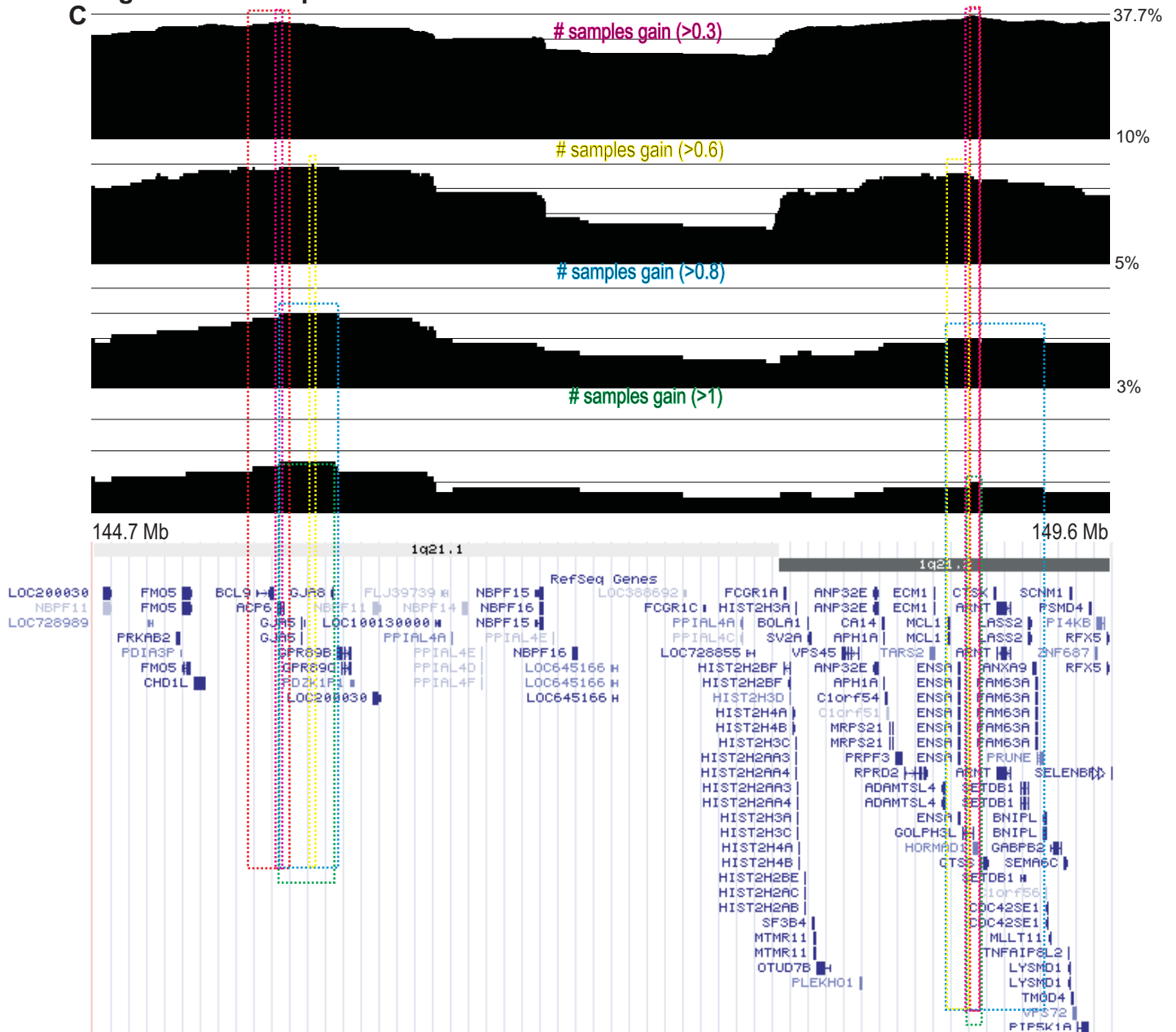

Supplement: Figure S7 — Gain on chr1. A. Frequency of gain on chr1 is shown at various amplitude thresholds. Note the different scales for each threshold. The GISTIC -log q value is plotted above, as is the extent of the broad GISTIC region identified (red bar). GISTIC peaks are indicated by arrows. Zoomed in views of 1p34 (B) and 1q21 (C). Each of the minimal peak frequency regions for each CN amplitude is shown by a coloured box. The GISTIC peak is indicated by the red box. Below are shown the genes from the UCSC genome browser. (0.07 MB PDF) [file pone.0011408.s007.pdf]

Figure S8

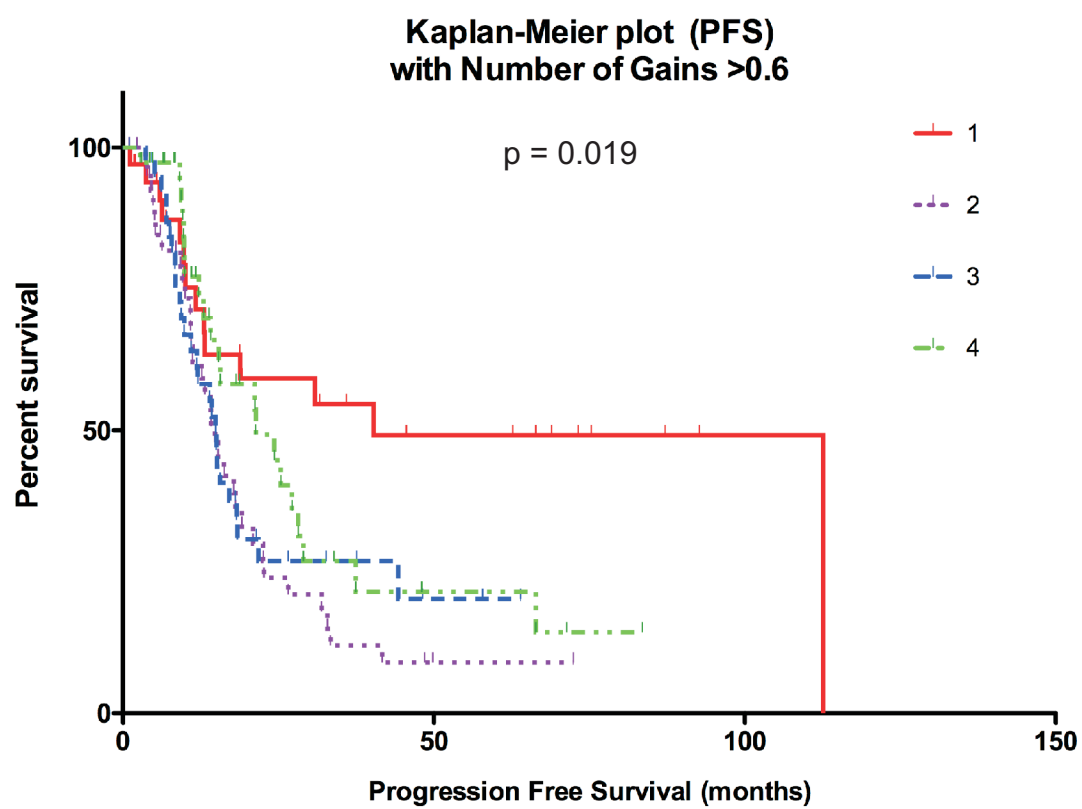

Supplement: Figure S8 — Survival analysis. A. Kaplan Meier plot of overall survival with samples divided into quartiles based on the number of gains >0.6 (log2). P = 0.045 after a Cox proportional hazard model analysis. 1, 0–18 segments; 2, 19–36 segments; 3, 37–60 segments; 4, >60 segments. B. Kaplan Meier plot of overall survival with residual macroscopic disease as a factor. (0.08 MB PDF) [file pone.0011408.s008.pdf]
