# Supplementary material for: Copy Number Analysis Identifies Novel Interactions Between Genomic Loci in Ovarian Cancer
Source: PLoS One. 2010 Sep 10;5(9):e11408. doi: 10.1371/journal.pone.0011408 (PMC2937017; doi:10.1371/journal.pone.0011408)
Supplement: Methods S1 — Supplementary methods. (0.06 MB DOC) [file pone.0011408.s009.doc]

**Supplementary Methods**

*1. Copy number array pre-processing and QC.*

A number of QC measures were undertaken as follows:

a) Genotype call rate. All samples were required to have at least an 85% call rate. For 500K arrays, if one array failed, then both were excluded.

b) Genotype concordance. All tumour samples were tested for concordance to all normal samples to ensure each were correctly matched. All samples were also compared to all other samples to ensure there were no duplicates.

c) Visual inspection of normal samples. Following copy number normalisation, all normal samples were visually inspected to ensure none had large scale alterations that might suggest a mix-up between normal and tumour. In this instance, both normal and tumour were removed from analysis. Normal samples were also excluded for high data noise that negatively impacted on the tumour copy number as compared to a bulk baseline.

d) Visual inspection of tumours. We checked each sample’s copy number data visually to remove poor performing samples, due to noise or data “waves”.

As some of the steps involved in the normalisation of the arrays are multi-array methods, normalisation steps were redone after the removal of poor quality samples. As the 50K data set had been fully published elsewhere we did not QC this further here.

Samples passed QC measures as follows:

|  | 250K Sty arrays (Japan) | 500K | SNP6.0 | TCGA |
| --- | --- | --- | --- | --- |
| Start: | 27 tumour, 24 normal | 32 tumour and normal | 83 tumour, 50 normal | 163 tumour, 161 normal |
| Genotype call rate: | 1 normal failed | 4 failed | All pass | 1 failed |
| Genotype concordance: | All pass | All pass | All pass | All pass |
| Visual normals: | 4 failed | All pass | 2 failed | 2 failed |
| Visual/MAPD tumours: | 1 failed | 1 failed | All pass | 5 fail |
|  | 23 passed of which 18 paired | 27 paired pass | 83 pass, of which 48 paired | 157 pass, of which 146 paired |

*2. Copy number thresholds*

Each array platform has different characteristics and may display batch effects. Tissue samples from different laboratories have been processed slightly differently, for example with/without microdissection, different DNA extraction procedures. Also each platform was normalised independently. To take this variation into account, we assessed whether a different threshold for each data set, based on characteristics of the data, would be better than a common threshold. We used a method similar to that employed previously by TCGA analysis of the glioblastoma data set [48], in which the first increase in frequency of segment mean outside of the central modal peak(s) was selected as a cutoff (Table below). However, when we compared the frequency plots using platform-specific and a generic cut-off, there were very few differences observed, thus we elected to continue with only a common threshold for all platforms.

| Data Set | Loss | Gain |
| --- | --- | --- |
| 50K Aus | -0.17 | 0.3 |
| 250K Jap | -0.13 | 0.13 |
| 500K Aus | -0.19 | 0.25 |
| SNP6 Aus | -0.16 | 0.3 |
| TCGA | -0.13 | 0.28 |

*3. Analysis of associations*

We defined the regions to analyse using GISTIC on the TCGA data set as described in the main text. This analysis called 61 regions of gain and 62 regions of loss, all of which were treated separately. Each minimal peak region was small and no sample had a copy number breakpoint within the region. Thus, to score a sample, each minimal peak region was queried in each sample using the segmented data. For example, on chromosome 20q11 the minimal peak region of gain was 29.862 - 29.907 Mb. A list of segmented gains (>0.3 log_2_ ratio) was queried to determine whether each sample had a gain at that region, in which case it was scored as “1”. If a sample did not have a gain at that region (i.e. was CN neutral or CN loss), it was scored as a “0”. Similarly, for peaks that were identified as a region of loss, samples were scored “1” if they had a loss at the region and “0” if they did not (i.e. were CN neutral or CN gain). Thus, each peak region from GISTIC was only scored positively for each sample if in the same direction as the original peak.

As described in the methods, we then performed an association analysis, looking for those pairs of regions where there were more samples that had both more aberrations than expected by chance (positive association) or less (negative association) given the overall frequency of each aberration in all samples. The direction of the association (positive or negative) did not affect the p-value but was determined separately. The test for association was blind to whether the peak was originally identified as a gain or a loss.

**Table S1 Full list of samples**

**Table S2 Full list of frequent aberrations and genes**

**Table S3 Full list of associated aberrations**

**Table S4 Expression of genes within associated aberrations**

**Table S5 Clinical correlations of aberrations**

**Figure S1. Comparison of samples run on different array platforms.**  **A** Overall frequency plot of gains and losses for SNP6 TCGA (n=157), SNP6 PMCC (n=83), 500K PMCC (n=27), 250K Japanese (n=23) and 50K PMCC (n=108). All platforms used the same log_2_ threshold of ±0.3 **B. Hierarchical clustering of samples**. Samples were scored as positive (red) or negative (blue) for gains and losses identified in all SNP6 samples by GISTIC. Sample source, histological subtype and grade are indicated in colour at top. There is no apparent grouping by array source that would suggest a batch effect of the arrays, apart from the Japanese 250K samples (grade unknown), which tend to have few alterations and cluster with the low grade endometrioid samples at the left hand side of the dendrogram.

**Figure S2. Gain on 3q.** **A**. Frequency of gain on 3q is shown at various amplitude thresholds. Note the different scales for each threshold. The GISTIC -log q value is plotted above, as is the extent of the broad GISTIC region identified (red bar). GISTIC peaks are indicated by arrows. **B**. Zoomed in view of 3q26. Each of the minimal peak frequency regions for each CN amplitude are shown by coloured boxes. The GISTIC peak is indicated by the red box. Below are shown the genes from the UCSC genome browser.

**Figure S3. Gain on chr19. A**. Frequency of gain on chr19 is shown at various amplitude thresholds. Note the different scales for each threshold. The GISTIC -log q value is plotted above, as is the extent of the broad GISTIC region identified (red bar). GISTIC peaks are indicated by arrows. Zoomed in views of 19p13 (**B**) and 19q12 (**C**) Each of the minimal peak frequency regions for each CN amplitude are shown by coloured boxes. The GISTIC peak is indicated by the red box. Below are shown the genes from the UCSC genome browser.

**Figure S4. Gain on 11q. A**. Frequency of gain on 11q is shown at various amplitude thresholds. Note the different scales for each threshold. The GISTIC -log q value is plotted above, GISTIC peaks are indicated by arrows. Asterisks – 3 possible amplicons **B**. Zoomed in view of 11q13-14. Each of the minimal peak frequency regions for each CN amplitude are shown by coloured boxes. The GISTIC peak is indicated by the red box. Below are shown the genes from the UCSC genome browser.

.

**Figure S5. Gain on 8q. A**. Frequency of gain on 8q is shown at various amplitude thresholds. Note the different scales for each threshold. The GISTIC -log q value is plotted above, as is the extent of the broad GISTIC region identified (red bar). GISTIC peaks are indicated by arrows. **B**. Zoomed in view of 8q24. Each of the minimal peak frequency regions for each CN amplitude are shown by coloured boxes. The GISTIC peak is indicated by the red box. Below are shown the genes from the UCSC genome browser.

**Figure S6. Gain on chr20. A**. Frequency of gain on chr20 is shown at various amplitude thresholds. Note the different scales for each threshold. The GISTIC -log q value is plotted above, as is the extent of the broad GISTIC region identified (red bar). GISTIC peaks are indicated by arrows. Zoomed in views of 20q11 (**B**), 20q13.2 (**C**) and 20q13.33 (**D**). Each of the minimal peak frequency regions for each CN amplitude are shown by coloured boxes. The GISTIC peak is indicated by the red box. Below are shown the genes from the UCSC genome browser. **E**. **Gain on 12p.** Frequency of gain on chr12 is shown at various amplitude thresholds. Note the different scales for each threshold. The GISTIC -log q value is plotted above, as is the extent of the broad GISTIC region identified (red bar). GISTIC peaks are indicated by arrows. **F**. Zoomed in view of 12p, with various genes indicated.

**Figure S7. Gain on chr1. A**. Frequency of gain on chr1 is shown at various amplitude thresholds. Note the different scales for each threshold. The GISTIC -log q value is plotted above, as is the extent of the broad GISTIC region identified (red bar). GISTIC peaks are indicated by arrows. Zoomed in views of 1p34 (**B**) and 1q21 (**C**). Each of the minimal peak frequency regions for each CN amplitude are shown by coloured boxes. The GISTIC peak is indicated by the red box. Below are shown the genes from the UCSC genome browser.

**Figure S8. Survival analysis.** **A**. Kaplan Meier plot of overall survival with samples divided into quartiles based on the number of gains > 0.6 (log_2_). P = 0.045 after a Cox proportional hazard model analysis. 1, 0-18 segments; 2, 19-36 segments; 3, 37-60 segments; 4 - >60 segments. **B**. Kaplan Meier plot of overall survival with residual macroscopic disease as a factor.
